# DNA Ligase 1 is an essential mediator of sister chromatid telomere fusions in G2 cell cycle phase

**DOI:** 10.1093/nar/gky1279

**Published:** 2018-12-21

**Authors:** Kate Liddiard, Brian Ruis, Yinan Kan, Kez Cleal, Kevin E Ashelford, Eric A Hendrickson, Duncan M Baird

**Affiliations:** 1 Division of Cancer and Genetics, School of Medicine, Cardiff University, Heath Park, Cardiff CF14 4XN, UK; 2 Department of Biochemistry, Molecular Biology, and Biophysics, University of Minnesota, Minneapolis, MN 55455, USA

## Abstract

Fusion of critically short or damaged telomeres is associated with the genomic rearrangements that support malignant transformation. We have demonstrated the fundamental contribution of DNA ligase 4-dependent classical non-homologous end-joining to long-range inter-chromosomal telomere fusions. In contrast, localized genomic recombinations initiated by sister chromatid fusion are predominantly mediated by alternative non-homologous end-joining activity that may employ either DNA ligase 3 or DNA ligase 1. In this study, we sought to discriminate the relative involvement of these ligases in sister chromatid telomere fusion through a precise genetic dissociation of functional activity. We have resolved an essential and non-redundant role for DNA ligase 1 in the fusion of sister chromatids bearing targeted double strand DNA breaks that is entirely uncoupled from its requisite engagement in DNA replication. Importantly, this fusogenic repair occurs in cells fully proficient for non-homologous end-joining and is not compensated by DNA ligases 3 or 4. The dual functions of DNA ligase 1 in replication and non-homologous end-joining uniquely position and capacitate this ligase for DNA repair at stalled replication forks, facilitating mitotic progression.

## INTRODUCTION

DNA ligase I (LIG1) is one of three identified human DNA ligases involved in multiple essential intracellular pathways (1,2). Whilst DNA ligase 3 (LIG3) and 4 (LIG4) have long been ascribed functions in non-homologous end-joining (NHEJ) repair (3), LIG1 has conventionally been associated with DNA replication (4–7). During the synthesis (S) phase of the mitotic cell cycle, the genome is replicated such that it can be partitioned equally amongst the progeny during the mitotic (M) phase. Leading and lagging strands of the double helix are differentially synthesized, with the nascent DNA derived from the lagging strand is produced as a series of short (100–300 nucleotide) Okazaki fragments (8) that require reassembly by LIG1. Consequently, LIG1 function is intimately linked with proliferative capacity (9) and its upregulated expression has been documented in human cancers (10). Intriguingly, mutations that compromise LIG1 activity are also affiliated with cancer (11–13). Specifically, a patient presenting with developmental delays, immune deficiency and lymphoma was identified as having compound heterozygous mutations in *LIG1* that severely reduced functional capacity. Fibroblasts derived from this patient demonstrated a range of DNA processing defects, including delayed ligation of replication intermediates, replication fork errors, enhanced sensitivity to DNA damaging agents (14) and hyperactivation of sister chromatid exchanges (15). Subsequent research has positioned LIG1 at the interface of interdependent DNA processing and repair pathways, including long-patch base-excision repair (LP-BER) (16), nucleotide excision repair (NER) (17), mismatch repair (MMR) (18) and, more recently, non-homologous end-joining (NHEJ) (19–21). Furthermore, advances in high-resolution molecular exploration of nucleic acid metabolism have delineated an ever-growing complexity of pathway interactions and defined novel subcategories of DNA repair in which LIG1 may also be pivotal (22). Collectively, these studies highlight the critical importance of this ligase in the DNA repair processes that safeguard genome integrity.

For intelligently targeted therapeutic intervention (23), it is imperative to achieve clear separation of function between the DNA ligases and to more precisely understand the diversity, hierarchy and restrictions associated with the processes they coordinate. Notably, LIG3 and LIG1 appear functionally interchangeable in some experimental models (20,24–27) and genetic targeting has revealed a redundancy that permits viability with the solitary absence of either enzyme (28,29). The catalytic core of LIG1 and LIG3 is highly-conserved, suggesting that diversification of function is conferred by the unique N- and C-termini of the respective ligases and the particular protein mediators with which they interact (1). Intracellular temporal and spatial segregation of LIG1 and LIG3 (30) may reinforce functional disjunction and subtle differences in ligation kinetics and avidity (31,32) may dictate pathway selection under competitive conditions (33). Importantly, we have already documented a non-redundant role for LIG3 in the specialized DNA repair activity that permits cellular escape from a telomere-driven crisis (34). Thus, whilst LIG1 and LIG3 may have overlapping functional spectra, it is apparent that they also independently-regulate distinct processes.

Telomere fusions represent a mutagenic DNA repair response to the recognition of shortened or damaged and deprotected chromosome ends as double-strand breaks (DSBs). The recombination of sister chromatid or heterologous chromosomal telomeres is mediated by NHEJ to produce dicentric chromosomes that can precipitate global genomic instability through progressive breakage-fusion-breakage cycles or more acute genetic fragmentation under the pressure of persistent mitosis (35,36). Fusions are rare in normal proliferating or senescent cells but can be detected with increasing frequency during crisis or in response to targeted DSBs (21,37). Significantly, these events have been reported in several malignancies in association with oncogenic transformation (38–40). The conspicuous emergence of telomere fusions and the express involvement of NHEJ components in their formation presents an unparalleled forum within which to rigorously investigate the relative activities of distinct DNA ligases. We formerly uncovered the potential engagement of LIG1 in telomere fusions that arise in absence of operational LIG3- and LIG4-mediated alternative and classical NHEJ (A-NHEJ and C-NHEJ), respectively (21). By targeting a DSB to a specific telomere-proximal sequence, we were able to induce and perform the detailed molecular characterization of a diversity of inter- and intra-chromosomal telomere fusions in cells differentially compromised for DNA repair. Although inter-chromosomal fusions were rarely recovered from cells lacking both LIG3 and LIG4, we determined an unexpected proficiency of these *LIG3^−/−^:LIG4^−/−^* cells for intra-chromosomal sister chromatid fusions that implicated the remaining ligase, LIG1, as being competent and sufficient to effect these particular telomere recombinations.

In this study, we have exploited this bespoke DSB-induced telomere fusion strategy to confirm and further resolve the precise requirement for LIG1 in sister chromatid telomere fusion events that precede chromosome condensation and partition of the genome in mitosis. These studies demonstrate that LIG1 is critically employed in DNA repair at specific stages of the cell cycle when homology-dependent recombination (HDR) activity is incompatible with the coordinated resolution of chromosomal damage (41–44) and viable cytokinesis (45).

## MATERIALS AND METHODS

### Cell culture

All cells were routinely cultured at 37°C in 5% CO_2_, screened for the absence of mycoplasma and authenticated by functional or expression assays appropriate to their specific genetic backgrounds.

HCT116 cell lines were cultured in McCoy's 5A medium supplemented with 10% (v/v) fetal calf serum, 1 × 10^5^ IU/l penicillin, 100 mg/l streptomycin, and 2 mM glutamine. Antibiotic selection agents were also added as required for long-term cultures (1 μg/ml puromycin for *LIG1^−/flox:CreERt2^*, 1 μg/ml puromycin and 2.5 μg/ml G418 for the *LIG3^−/−^:LIG4^−/−^:LIG1^−/flox:CreERt2^*). Single cell suspensions were stained with 30 μg/ml acridine orange and 100 μg/ml DAPI (4′,6-diamidine-2′-phenylindole dihydrochloride) to count viable cells and determine mean cell diameters using the Chemometec NC-3000 image cytometer.

Translocation of the CreERt2 transgenic fusion protein was achieved by an 18 to 24 h treatment with 1 μM 4-OHT (4-Hydroxytamoxifen; Sigma H6278) diluted 1/10000 with supplemented McCoy's 5A from a 1 mg/ml stock in methanol for application to cells. Methanol carrier controls were prepared using the same 1/10000 dilution with medium.

### Construction of *LIG1^−/flox:CreERt2^* HCT116 cell line

**Figure 1.**
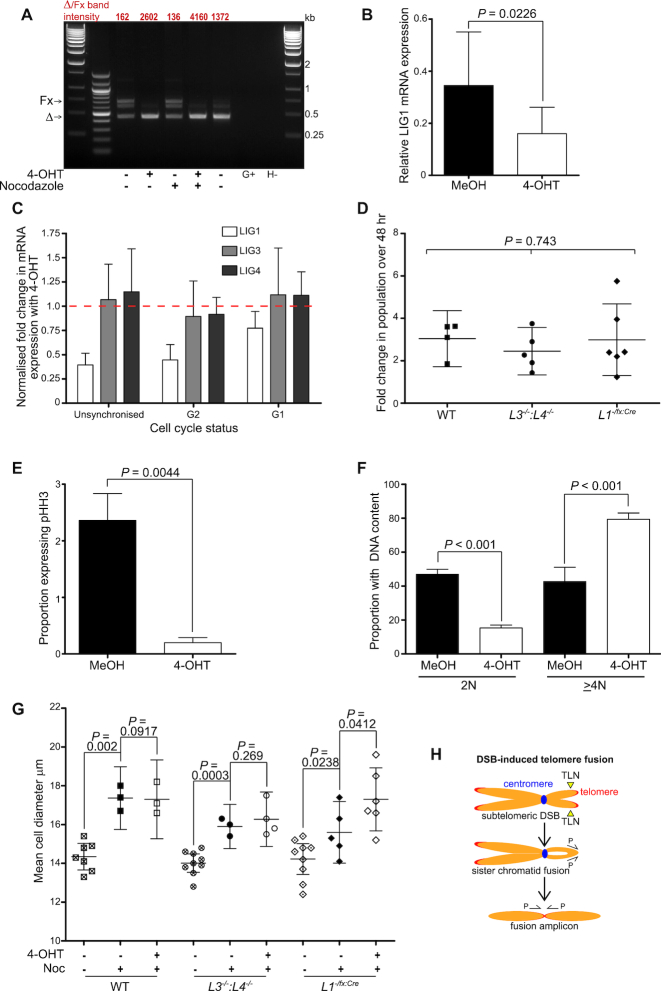
*LIG1* deletion results in cell cycle arrest and morphological changes. (**A**) Resolution of *LIG1* allelic recombination in 4-OHT and nocodazole-treated (G2-arrested) *L1^−/fx:Cre^* cells by agarose gel electrophoresis of genomic PCR products. Deleted/‘floxed’ (Δ/Fx) allelic ratios (×10^−2^) are indicated above the sample lanes. No amplification from a WT HCT116 control (G+) or water blank (H–) was detected. (**B**) Quantification of the reduction in mean relative LIG1 mRNA expression (Ex22_F and Ex23_R primers) in *L1^−/fx:Cre^* cells following 24 h treatment with 4-OHT from four replica experiments with 95% confidence intervals. Statistical significance was evaluated using a paired one-tailed *t*-test. (**C**) The specific down-regulation of LIG1 mRNA expression (Ex22_F and Ex23_R primers) by 4-OHT treatment over 24 h in unsynchronized or cell cycle G2- or G1-arrested *L1^−/fx:Cre^* cells from 3 replica experiments is depicted as a bar chart of mean fold change in expression with 95% confidence intervals. The dotted line illustrates a fold change of 1.0 (no change). (**D**) Comparison of WT, *L3^−/−^:L4^−/−^* and *L1^−/fx:Cre^* mean fold changes in 48 h post-thaw cell counts with 95% confidence intervals from at least four independent experiments assessed by Kruskal–Wallis one-way ANOVA. (**E**) 24 h treatment of treatment of *L1^−/fx:Cre^* cells with 4-OHT causes cell cycle arrest and loss of mitotic G2/M phase phospho-histone H3 (pHH3)-expressing populations determined by flow cytometry. Mean data from 5 independent experiments with 95% confidence intervals displayed and assessed by paired sample one-tailed *t*-test. (**F**) Mean proportions of *LIG1^−/flox:CreERt2^* cells with diploid (2N) and tetraploid (4N) DNA content from four independent experiments are plotted with 95% confidence intervals and paired one-tailed *t*-tests between 2N and 4N DNA content fractions. (**G**) Changes in mean cell diameter induced by 24 h MeOH (black shapes) or 4-OHT (white shapes) treatment of nocodazole G2-arrested WT, *L3^−/−^:L4^−/−^* and *L1^−/fx:Cre^* cells from three to nine independent experiments are shown as mean values with 95% confidence intervals. Unsynchronized untreated samples are displayed as hatched shapes. Unpaired one-tailed T-tests between appropriate samples were performed. (**H**) Illustration of TALEN-induced (TLN) subtelomeric DSB resulting NHEJ-mediated fusion of replicated and cohered sister chromatids in G2 phase of the cell cycle. A single orientation primer (P) can be used to amplify these head-head sister chromatid fusions for Southern hybridization or sequence analyses.

Cre recombinase loxP recognition sites (illustrated as filled arrowheads in Supplementary Figure S1A) were introduced flanking exon 23 of human *LIG1* in a wild-type (WT) HCT116 cell line using the adeno-associated viral promoter trapping vector (pAAV-LIG1-SEPT). Sequential rounds of targeting and Cre recombination resulted in a single clone harbouring one recombined *LIG1* deletion allele and one ‘floxed’ allele retaining the flanking loxP sites (HCT116 *LIG1^−/flox^*). A Cre-oestrogen receptor transgene (CreERt2) was subsequently added via lentiviral integration, permitting conditional recombination of the remaining *LIG1* allele at exon 23 by a transient 20 to 24 h exposure to 1 μM 4-OHT to produce a biallelic functional deletion of *LIG1*. Consequently, any resultant *LIG1* transcripts lack sequence encoding both the C-terminal DNA-binding and ligase domains. Correctly targeted clones were identified by PCR screening of genomic DNA. Validation of the Cre-mediated recombination of the single intact *LIG1* allele was performed by genomic PCR using primers designed to amplify and distinguish ‘floxed’ and deleted alleles: LIG1_LOXP_SF 5′-ATGTCCTGTTCGCCTCAC-3′; LIG1_LOXP_SR2 5′-AGGGTACACAGTGGAGTC-3′. Representative analyses are shown in Figure 1A. Confirmation of reduced LIG1 mRNA and protein expression following 4-OHT-induced allelic recombination is shown in Figure 1B, C and Supplementary Figures S1B, C. Figure 1E, F and Supplementary Figures S1D–F display the functional cell cycle arrest achieved upon 4-OHT-induced *LIG1* deletion.

### Construction of a *LIG3^−/−^:LIG4^−/−^:LIG1^−/flox:CreERt2^* HCT116 cell line

In order to generate a *LIG1* conditional knockout in HCT116 *LIG3^−/−^:LIG4^−/−^* double knockout cells that maintain essential expression of mitochondrial LIG3 (21), CRISPR/Cas9-mediated gene targeting was used (Supplementary Figure S4A). Briefly, a double strand DNA (DSB) in exon 23 of the *LIG1* gene was generated by Cas9 using the sgRNA 5′-GTAGGTGGCATCAACATCCA-3′. This was accomplished using a CRISPR/Cas9-t2A-GFP co-expression vector (PX458) along with a double stranded rAAV plasmid donor containing homologous sequences 5′ and 3′ to a ‘floxed’ *LIG1* exon 23 bearing a silent mutation that interrupts the PAM sequence for the gRNA. Upon screening 21 candidate clones, one (#15) was identified that had a single correctly ‘floxed’ *LIG1* allele with the other allele acquiring a deletion within exon 23 that resulted in a null allele. To generate the conditional biallelic *LIG1* null line, a PiggyBac CreERt2 Puro expression vector was randomly integrated by co-transfection with a PiggyBac transposase expression vector. The transfected cells were selected using 1 μg/ml puromycin and a total of five puromycin-resistant clones (including C15-5 and C15-9) were further characterized. Initial functional complementation was demonstrated by culturing the cells in the presence or absence 20 nM 4-OHT for 24 h, followed by 3 days in 10 nM 4-OHT before extraction of genomic DNA for confirmation by genomic PCR. Validation of the Cre-mediated recombination of the single intact *LIG1* allele was performed by genomic PCR using primers designed to amplify and distinguish ‘floxed’ and deleted alleles: LIG1 Ex22_F 5′-CTGGAGCAGTCAGTGAAAGG-3′; LIG1 Int23_R 5′-GGCTGTTCTCCATCAGAACTC-3′. In subsequent experiments, Cre-mediated *LIG1* recombination was induced by a transient 20 to 24 h exposure to 1 μM 4-OHT to produce a biallelic functional deletion of *LIG1*. Representative analyses are shown in Supplementary Figures S4C(ii) and D(i). S4C(iii) demonstrates the reduction in LIG1 mRNA expression and Supplementary Figure S1C illustrates the consequent diminution in LIG1 protein achieved upon 4-OHT-induced *LIG1* deletion in the *LIG3^−/−^:LIG4^−/−^* background. Figure 5A confirms the functional cell cycle resulting from the LIG1 depletion.

### Inhibitors

The LIG1 inhibitor, L82, was a kind gift of Dr Alan E. Tomkinson and Dr Tim Howes of the University of New Mexico, USA (23,46). L82 was reconstituted to 10 mM using DMSO and stored in aliquots at −80°C. Stock solutions were further diluted to 5 and 50 μM in cell culture medium for use in the experiments indicated.

For enrichment of cells in the G2 cell cycle phase, cultures were treated with the microtubule disruptor, nocodazole (Sigma M1404) at 50 ng/ml. Serial dilutions were prepared in PBS and supplemented medium from a 1 mg/ml stock in DMSO. Carrier controls were prepared using the same serial dilutions of DMSO. Cells were typically pre-treated for 18 to 24 h before nucleofection experiments.

For enrichment of cells in the G1 cell cycle phase, cultures were treated with the iron chelator DNA replication inhibitor, l-mimosine (Sigma M0253) at 200 μM diluted in supplemented culture medium. Cells were typically pre-treated for 18 to 24 h before nucleofection experiments.

Confirmation of inhibition was confirmed by cell cycle analyses using a Chemometec NC-3000 image cytometer or Accuri C6 flow cytometry to detect cellular DNA content and proportions of G2/M mitotic cells. More than 5000 events were routinely collected for analyses of sample populations.

Where experiments required *LIG1* recombination of G2 phase cells, cells were treated with nocodazole for 4–6 h before addition of 4-OHT and continued culture for a further 20 h prior to nucleofection. Post-nucleofection, cells were re-treated with cell cycle inhibitors after a 30 min recovery period and re-dosed in fresh medium after 24 h for samples to be harvested at 48 h.

### Cell cycle analyses

Cell cycle analyses of fixed cells were performed using either the Accuri C6 flow cytometer or the Chemometec NC-3000 image cytometer depending on experimental design.

For NC-3000 assays, cells were fixed with 70% ethanol for 2 to 24 h at −20°C prior to permeabilization with 0.1% Triton X-100 and incubated with 1 μg/ml DAPI DNA stain for 5 min at 37°C. Exported FCS3 files were processed using Flowing software to delineate cell cycle phases and determine proportions of cells in each fraction.

For Accuri C6 flow cytometry, cells were fixed with 1% formaldehyde for 10 min at 37°C before being permeabilized with methanol (90% final concentration) at −20°C for 2 to 24 h. Cells were pelleted and washed with PBS before being incubated with 50 μM DRAQ5™ or 125 ng/ml DAPI DNA staining for 30–60 min in the dark at room temperature. More than 5000 events were collected with the Accuri C6 flow cytometer and cell cycle distributions were determined from histograms generated using the Accuri C6 analysis software.

### Detection of pHH3- and γH2AX-expressing populations by flow cytometry

To determine the fractions of actively dividing G2/M mitotic cells, the Cell Signaling Technology rabbit monoclonal antibody, anti-phospho-histone H3 (Ser10) clone D2C8, conjugated with Alexa Fluor® 488 or Alexa Fluor® 647 was used at 1/50 dilution for <1 × 10^6^ cells.

To measure the DNA damage response, the BD Biosciences mouse monoclonal antibody, (N1-431) anti-γH2AX PerCP-Cy5 (BD564718), was used at 1/20 dilution for <1 × 10^6^ cells.

Cells were harvested 24 h post-nucleofection and single cell suspensions were fixed with 1% formaldehyde and permeabilized with methanol to 90% final volume for 2 to 24 h at −20°C. Samples were blocked with either 5% rabbit or 2% foetal calf serum in PBS, then washed by centrifugation and incubated with antibodies indicated for 45 min in the dark at room temperature. Detection of expressing cells (signal intensity threshold set at 10^4^) was carried out using the Accuri C6 flow cytometer collecting >5000 events.

### Flow cytometry of isolated nuclei

To confirm the nocodazole-induced increase in cellular DNA content was due to cytokinesis block rather than formation of multinuclear or fused cells, analysis of DNA content in isolated nuclei was performed by flow cytometry. Nuclei were isolated according to the protocol established by Zeng *et al.* (47). Briefly, 1 × 10^6^ cells were pelleted and resuspended in PBS for accurate assessment of mean cell diameter and viability using the Chemometec NC-3000 image cytometer ahead of nuclear isolation. Cells were lysed in buffer containing 10 mM Tris–HCl pH7.4, 10 mM NaCl, 3 mM MgCl_2_ and % IGEPAL CA-630 on ice for 20 min. Liberated nuclei were pelleted by centrifugation at 500 × *g* for 20 min at 4°C. Completion and purity of lysis and extraction were verified by determination of expected reduction in particle diameter and viability using elevated concentrations of acridine orange (80 μg/ml) for more sensitive detection of smaller nuclei using the NC-3000. Purification was repeated by additional lysis or filtration through 20 μm sterile CellTrics filters where necessary.

Purified nuclei were fixed with 1% formaldehyde and permeabilized with methanol at 90% final concentration for 24 h at −20°C before antibody staining. Single nuclei suspensions were serum-blocked (2% FCS) and incubated for 30 to 45 min in the dark at room temperature with 1/50 dilution of Cell Signaling Technology rabbit monoclonal antibody anti-phospho-histone H3 (Ser10) clone D2C8 conjugated with Alexa Fluor^®^ 488. Cells were washed in PBS and counterstained with 50 μM DRAQ5™ for 30 min in the dark at room temperature. Detection of phospho-histone H3 (pHH3)-expressing cells (signal intensity threshold set at 10^4^) and determination of DNA content was carried out using the Accuri C6 flow cytometer collecting >5000 events.

### 17p and 16p/21q (21q) subtelomere TAL effector nucleases

TAL Effector Nucleotide Targeter 2.0 software (Cornell University) (48) was used to design TAL Effector Nuclease (TALEN) pairs to cleave human subtelomeric sequence at targeted single nucleotide locations. One pair (17p TLN) was designed to target the unique 17p subtelomere sequence 32 bp centromeric to start of the telomere repeat array. One pair (21q TLN) was designed to target sequence 1.5 kb from the start of the telomere repeat arrays of the homologous 16p- and 21q-related telomere families. TALEN pairs and surrogate reporters for targeting validation were custom-synthesized by LabOmics S.A. and endotoxin-free transfection-quality plasmid preparations were purified from Stbl3 competent cells (Invitrogen) using Nucleobind Xtra Midi Plus kits (Macherey-Nagel). Plasmid identity was confirmed by *Xba*I restriction enzyme mapping and Sanger DNA sequencing.

### 17p and 16p/21q subtelomere TALEN transfections

Subconfluent (50 to 80%) HCT116 cells were nucleofected with 2.5 μg of each 17p subtelomere-specific (17p TLN) or 16p/21q family subtelomere-specific (21q TLN) TALEN plasmid in batches of 1 × 10^6^ cells/cuvette in 100 μl supplemented nucleofection mix (Lonza SE Cell Line Kit L) using the Amaxa 4D nucleofector program DS-138. Following 10 min recovery post-nucleofection in unsupplemented RPMI at 25°C and 15 min incubation in complete McCoy's 5A at 37°C, transfection replicates were pooled for co-culture to eliminate inter-sample variation. Cells were plated into 6- or 12-well tissue culture plates and supplemented with the requisite experimental cell cycle inhibitors. Selection antibiotics and 4-OHT were not re-applied post-nucleofection. Cells were harvested at 24 (t24) or 48 (t48) h post-nucleofection, with re-application of cell cycle inhibitors at the 24 h time point to ensure cell cycle arrest was sustained for 48 h. Harvested cells were pelleted and counted for use in downstream analyses as indicated.

Control (no DNA) or GFP plasmid transfections were performed in each experiment to determine transfection efficiency as well as the background level of DNA damage and spontaneous telomere fusion.

### Determination of transfection efficiency

1 × 10^6^ cells were nucleofected with 1 μg of a GFP expression plasmid (Lonza pmaxGFP) and harvested 24 h later for analysis of GFP expression in viable cells using the Chemometec NC-3000 image cytometer. Representative single cell samples were aliquoted and supplemented with Hoechst 33342 at a final concentration of 10 μg/ml. Samples were incubated for 15 min at 37°C before propidium iodide was added to a final concentration of 10 μg/ml and GFP expression in viable cell populations was measured and extrapolated to the sample bulk as a transfection efficiency percentage.

### RNA extraction and RT-PCR:

Total RNA was extracted using the Analytik Jena InnuPrep RNA Mini Kit with elution into nuclease-free water. RNA concentrations were measured using a nanodrop spectrophotometer and 1 μg purified RNA was reacted in the presence or absence of reverse transcriptase using the Applied Biosystems High Capacity cDNA RT kit to distinguish true cDNA amplification from residual genomic DNA template. Equal volumes of cDNA were subjected to 32 cycle RT-PCR using intron-spanning primers unique to each experimental tester gene and an annealing temperature of 60°C.

RT-PCR primers used in this study and the relative amplicon sizes are listed as Supplementary Table S1.

Amplicons were resolved by agarose (1% weight/volume) gel electrophoresis with ethidium bromide DNA staining and images were captured using a Vilbert Lourmat Ebox VX2 gel documenter. 1 kb and 100 bp DNA ladders were routinely run for sizing of amplicons. Quantification of amplicon signal intensity with local background correction was performed using ThermoFisher MyImageAnalysis software and normalized to values calculated for the constitutively-expressed Tyrosine 3-Monooxygenase/Tryptophan 5-Monooxygenase Activation Protein Zeta (*YWHAZ*) control gene within the same samples. Normalized gene expression values are presented as tester/*YWHAZ* signal intensity.

### Western blotting

HCT116 wild-type (WT), *LIG3^−/−^:LIG4^−/−^, LIG1^−/flox:CreERt2^*, parental clone C15 *LIG3*^−/−^*:LIG4*^−/−^*LIG1*^−^*/flox* and *LIG3^−/−^:LIG4^−/−^:LIG1^−/flox:CreERt2^* clones C15-5 and C15-9 were cultured in the presence of 1 μM 4-OHT or methanol (MeOH) carrier control for 24 h before 2 × 10^6^ cells of each sample were pelleted and snap frozen for protein extraction. Cells were lysed with RIPA buffer supplemented with protease inhibitors and proteins harvested by centrifugation. Lysates were quantified using Sigma Advanced Protein Assay reagent in comparison with a BSA protein standard curve read at OD_600_. 10 μg each sample lysate was boiled in 6X Laemmli's loading dye with β-mercaptoethanol and run on a 7.5% Tris-glycine gel for 80 min at 100 V. Transfer to PVDF membranes was at 70 V for 90 min. Membranes were blocked in 5% milk-TBST for 1 h at room temperature before incubation with primary antibodies diluted in 5% milk-TBST at 4°C overnight with agitation. Primary antibodies used in this study were: GeneTex rabbit polyclonal anti-C-terminal human LIG1 antibody (GTX102936) diluted 1/1000, Abcam mouse monoclonal anti-human LIG1 (amino acids 1-919) antibody [10H5] (ab615) diluted 1/3000 and Sigma rabbit polyclonal anti-C-terminal actin fragment antibody (A2066) diluted 1/1000. Amersham ECL secondary HRP-conjugated anti-rabbit (NA934) or anti-mouse (NA931) antibodies were used, as appropriate, at 1/10 000 dilution in 5% milk-TBST with incubation for 1 h at room temperature. Pierce™ ECL Plus Western Blotting Substrate was used for signal detection via exposure to X-ray film. Membranes were stripped using a mild stripping glycine-SDS protocol at room temperature before repeat blocking and immunoprobing. Total protein loading was visualized post-immunoprobing by staining membranes with 0.1% liquid Indian ink (Winsor and Newton) -PSBT overnight. Quantification of signal volume (intensity) from scanned images was performed using ThermoFisher MyImageAnalysis software. A composite normalization method was employed to calculate the fold change in aggregated LIG1 protein expression in 4-OHT-treated compared with MeOH-treated samples corroborated using two independent primary antibodies raised in different species to confirm antigen specificity. Sample protein equivalence was assessed by dual quantification of actin housekeeping and constitutive protein loading signal determined by immunoprobing and membrane staining.

### Genomic DNA extraction

Total genomic DNA from HCT116 was extracted using Sigma GenElute™ Mammalian Genomic DNA Miniprep Kits and eluted in nuclease-free water. DNA was quantified using a nanodrop spectrophotometer to confirm a consistent purity and DNA yield for use in genomic PCR, telomere fusion PCR and sequencing reactions.

### Genomic PCR

Cre-mediated recombination of *LIG1* exon 23 in *LIG1^−/flox:CreERt2^* and *LIG3^−/−^:LIG4^−/−^:LIG1^−/flox:CreERt2^* HCT116 cells was routinely assessed by genomic PCR using the primers detailed in Supplementary Table S1 and 100–200 ng sample genomic DNA. Defined positive control genomic DNA and negative control water blanks were included in every experiment.

Amplicons were resolved by agarose (1% weight/volume) gel electrophoresis with ethidium bromide DNA staining and images were captured using a Vilbert Lourmat Ebox VX2 gel documenter. 1 kb and 100 bp DNA ladders were routinely run for sizing of amplicons. Quantification of amplicon signal intensity with local background correction was performed using ThermoFisher MyImageAnalysis software.

### Telomere fusion PCR and Southern hybridization

Telomere fusion amplicons were generated from sample input genomic DNA (100 ng unless otherwise indicated) by multiplex long-range PCR using primers targeting the 17p, XpYp and 21q family of homologous telomeres (17p6, XpYpM and 21q1 primers, respectively; detailed in Supplementary Table S1) (36,49). This resulted in mixed pools of fusion amplicons with divergent molecular weights. A 2.9 kb constitutive amplicon band of known sequence identity is also detected with the 21q probe, but excluded from all fusion amplicon analyses. For standard experiments, three or four individual replicate reactions were typically resolved by 0.5% TAE agarose gel electrophoresis for detection on Southern blots using radiolabelled telomere-adjacent probes specific for each telomere end. To estimate telomere fusion frequency in each sample, the number of resolved non-constitutive fusion amplicons revealed by Southern blotting was summarized and divided by the mass in ng of input DNA since ploidy was variable amongst samples. Fusion frequencies are therefore expressed as events per ng rather than per diploid genome.

### Illumina MiSeq and HiSeq 2000 paired-end sequencing of 17p telomere sister chromatid fusions

17p TALEN-induced sister chromatid telomere fusions were amplified from G2-arrested HCT116 *LIG1^−/flox:CreERt2^* cells harvested 24 h post-nucleofection in 250 telomere fusion PCR replicates for Illumina MiSeq 300 cycle 150 bp paired-end sequencing. Random PCR wells were selected for cross-checking validation prior to the replicates for each sample being pooled and purified using Agencourt AMPure XP magnetic beads with elution in nuclease-free water. Aliquots of each sample were taken pre- and post-purification for a comparative assessment and to check purification efficiency. Sequencing of the G2-arrested *LIG3^−/−^:LIG4^−/−^* fusions was performed in collaboration with the Wales Gene Park using the Nextera XT sample prep kit.

Illumina HiSeq 2000 paired-end sequence data for 17p TALEN-induced telomere fusions derived from unsynchronized *LIG3^−/−^:LIG4^−/−^* cells at 48 h post-nucleofection (85 fusion PCR replicates) and *LIG3^−/−^* cells at 48 and 120 h (85 and 170 fusion PCR replicates, respectively) post-nucleofection was collected and explored in a former study (21). New analyses of the unsynchronized *LIG3^−/−^:LIG4^−/−^* 17p sister chromatid telomere fusion-mapping reads pertaining to the 48 hour time point only were conducted for the current study, in contrast with the pooled analyses of all 48 and 120 h data in the previous study (21).

Paired-end sequence read quality of each sample was checked prior to analysis using the Fastqc (v0.11.2; http://www.bioinformatics.babraham.ac.uk/projects/fastqc/) software package alongside in-house tools that assessed mapping success and insert size-range. All reads were trimmed and filtered with Trimmomatic (version 0.30 (50)) to remove sequencing adaptors and over-represented primer sequences. All scripts developed for the mapping and analysis of sequence data can be found on GitHub at https://github.com/nestornotabilis/GenomeResearch_2016_scripts.

Alignment of sequence reads, clone sequences and assembly contigs was performed with BWA-MEM version 0.7.4, arXiv:1303.3997). All mappings were performed with the default parameters save for the addition of the -M flag to mark shorter split hits as secondary for later filtering. Unless otherwise stated, all manipulations of the mapped outputs were performed using SAMtools version 0.1.19 (51). Sister chromatid 17p telomere fusion events were identified as discordant read-pairs mapping to the same strand in the same orientation towards the telomere indicative of head-to-head juxtapositions. Reads were first mapped to the 17p subtelomeric sequences and the SAMtools view command (samtools view -bF316) was applied to each output to filter for mapped paired-end reads where both the R1 and R2 tags were orientated on the same strand and oriented towards the telomere. A SAMtools mpileup command (samtools mpileup -ABd100000) was next applied to the filtered output to extract per-base coverage data. Indexed BAM files for all sample intra-chromosomal mapped and filtered read pairs were prepared for visualization in the Broad Institute Integrative Genomics Viewer (52).

### Junction analyses

Mapped 17p sister chromatid telomere fusions were analysed in high-resolution by BLAST alignment to define junction and recombination characteristics. The incidence of microhomology, insertions and resection from each individual fusion junction was determined manually. Microhomology was defined as ≥ 1 bp overlap in nucleotide (nt) usage in both fusion partners and events were subsequently categorized in terms of frequency, as well as the numbers of nt involved at each junction. Insertions (<50 bp) between apposed fusion partners were categorized as templated if ≥2 nt localized duplication or sequence similarity could be determined, or untemplated if unidentified by BLASTN mapping to any reference sequences. Deletion was measured back from the telomere-proximal 17p TALEN target site at 3024 bp from the 3′ terminal end of the 17p6 fusion amplifying primer. 17p chromatids extending beyond the TALEN cut site (either uncut or extended post-cleavage) were assigned a deletion value of 0.

### Quantification of nucleotide incorporation error rate

Illumina sequencing read pairs mapping to the 17p subtelomere were extracted and BLAST aligned to this reference to reveal nucleotide (nt) changes in the ‘centromeric’ (centromere-telomere orientation of single fusion amplicon sequenced 5′-3′) and ‘telomeric’ (telomere-centromere orientation) reads comprising each sister chromatid fusion event. The numbers of nt variant from the 17p subtelomere reference were normalized to read length adjusted for the exclusion of soft-clipped portions to generate the error rate/kb. All reads longer than 30 bp were included, but three SNP common to unsynchronized read pairs were excluded from the analysis.

### Statistical analyses

All statistical analyses, including one- and two-tailed *t*-tests, Mann–Whitney U-tests and Kruskal–Wallis ANOVA tests were performed using GraphPad Prism 6 and GraphPad QuickCalcs. Appropriate tests were selected according to whether data were collected as paired or independent experimental samples, whether data distributions were Gaussian or skewed and whether variance between comparators was equal. One-tailed *t*- and U-tests were applied where required to specifically assess a pre-defined direction of the biological effect under consideration. For all tests, a *P* value of <0.05 was considered statistically significant.

### Data access

BAM files containing trimmed and filtered data for the unsynchronized *LIG3^−/−^:LIG4^−/−^* HCT116 sample are available in the ArrayExpress database (www.ebi.ac.uk/arrayexpress) under accession number E-MTAB-3811.

BAM files containing trimmed and filtered data for the G2-arrested *LIG3^−/−^:LIG4^−/−^* HCT116 sample are available as a NCBI Bioproject (www.ncbi.nlm.nih.gov/bioproject), accession number PRJNA480939.

## RESULTS

### Depletion of LIG1 causes cell cycle arrest and altered morphology

We have reported the recovery of sister chromatid telomere fusions from HCT116 cells dually-deficient for the alternative- (A-NHEJ) and classical-non-homologous end-joining (C-NHEJ) nuclear-localized DNA ligases, LIG3 and LIG4, respectively (*LIG3^−/−^:LIG4^−/−^*) (21). The parity of these events with fusions amplified from cells individually deficient in either LIG3 or LIG4 suggested a redundancy amongst ligases for sister chromatid telomere fusion and led us to propose a role for LIG1 in the context of otherwise compromised C-NHEJ and A-NHEJ repair. To more precisely define the activity of LIG1 in sister chromatid telomere fusions, we utilized recombinant adeno-associated virus-mediated gene targeting to construct a heterozygous HCT116 *LIG1^−/flox:CreERt2^* cell line (*L1^−/fx:Cre^*) in which one allele had been inactivated and the other allele was ‘floxed’ by the introduction of loxP sites flanking exon 23 of the *LIG1* gene (Supplementary Figure S1A). Loss of exon 23 impedes the DNA binding function of LIG1 so that homozygous *LIG1^−/^*^*−*^ cells could not be propagated owing to the fundamental role of LIG1 in DNA replication. Exon 23 of the remaining *LIG1* allele was deleted by conditional activation of a Cre-oestrogen receptor fusion protein (CreERt2) with 4-hydroxytamoxifen (4-OHT) for 24 h, permitting fine temporal control over cellular depletion of functional LIG1. Appropriate allelic excision in 4-OHT-treated and not methanol (MeOH) carrier-control-treated cells was confirmed by comparative amplification of ‘floxed’ and deleted alleles by genomic PCR ahead of functional analyses (Figure 1A). Two independent HCT116 *LIG1^−/flox:CreERt2^* conditional recombination clones were generated from the original *LIG1^−/flox^* clone. For simplicity and consistency, data relating only to *LIG1^−/flox:CreERt2^* Clone 1 is presented in this study.

Induced recombination of the second *LIG1* allele resulted in a reproducible 2-fold reduction (*P =* 0.0226) in LIG1 mRNA expression within 24 h (Figure 1B, Supplementary Figure S1Bi). Recombination-associated down-regulation of LIG1 expression was determined under conditions of chemically-induced G1 (mimosine) and G2 (nocodazole) cell cycle arrest (verified in Supplementary Figure S1B(ii)), as well as in unsynchronized cells, confirming the suitability of the model for the investigation of LIG1 ligase function in NHEJ, unrestricted by cell cycle phasing. In contrast, expression of LIG3 and LIG4 mRNA was not substantially altered by the deletion of *LIG1* (Figure 1C), thus permitting separation of ligase functions. Using primers to detect mRNA transcripts derived from distinct exons of *LIG1*, we were able to confirm reduced expression of LIG1 both 3′ and 5′ of the Cre recombination locus at exon 23 (Supplementary Figure S1B(iii) in unsynchronized and G2-arrested cells in response to 4-OHT treatment. Conversely, transcripts corresponding to exons encoding N-terminal protein domains were detected at similar levels in 4-OHT-treated and untreated cells arrested in G1 conditions for 24 h. These results suggest a degree of LIG1 transcriptional feedback regulation following deletion of the catalytic and DNA binding domains critical for DNA replication and progression through cell cycle S-phase. Cells arrested prior to S-phase in G1 evade this replication stress engendered by ligase-incompetent LIG1 and maintain active transcription of this gene, albeit lacking exons encoding ligase functional domains following Cre-mediated recombination.

Significant reductions in LIG1 protein expression were also measured exclusively in cells harbouring a ‘floxed’ *LIG1* allele within 24 h of 4-OHT addition (Supplementary Figure S1C), indicating that the impact of *LIG1* deletion could be rapidly experienced within proliferating cells. Specifically, LIG1 expression was reduced 1.9-fold by 4-OHT-mediated *LIG1* deletion in *LIG1^−/flox:CreERt2^* cells compared with the stable expression in wild-type (WT) HCT116 (*P* = 0.0332). We were unable to measure expression levels of any N-terminal truncated LIG1 proteins in these experiments, although the decreases in mRNA derived from exons 4–5 (Supplementary Figure S1B(iii)) detected following exon 23 recombination suggest the possibility of inter-dependent regulation.

Over short-term experimental timescales, the *LIG1* haploinsufficiency effected by the constitutively-deleted allele in untreated *LIG1^−/flox:CreERt2^* clones did not significantly alter cellular growth rates (*P* = 0.743, Figure 1D) in comparison with wild-type (WT) or *LIG3^−/−^:LIG4^−/−^* (*L3^−/−^:L4^−/−^*) HCT116 cells. In contrast, the phenotypic consequences of deleting the remaining *LIG1* allele from HCT116 *LIG1^−/flox:CreERt2^* cells were acute and compelling. At 24 h post 4-OHT treatment, we determined a robust arrest in cell cycle progression of LIG1-depleted cells that manifested as an almost complete loss (11.8-fold reduction; *P* = 0.0044) of phospho-histone H3 (pHH3)-expressing G2/M phase mitotic cells (Figure 1E, Supplementary Figure S1D(i)) and an accumulation of tetraploid cells with ≥ 4N DNA content (Figure 1F, Supplementary Figure S1D(ii)). LIG1-depleted cells exhibited a 2.22-fold increase *(P* < 0.001) in post-replicated populations at the expense of a 3-fold reduction (*P* < 0.001) in proportions with normal diploid (2N) DNA content (Figure 1F). The 4-OHT-mediated cell cycle arrest was not reproducible in WT (Supplementary Figure S1D) and other DNA repair-deficient lines tested (Supplementary Figure S1E), indicating the effect was a specific consequence of LIG1 depletion and consistent with LIG1 being required for the efficient maturation of mitotic DNA replication intermediates (53,54). Ploidy increases were not caused by multinucleation since results using isolated nuclei (Supplementary Figure S1D(ii)) paralleled those obtained with whole cells (Supplementary Figure S1F). The expansion of cellular fractions with ≥ 4N DNA content following LIG1 depletion reflects both the retarded lagging strand DNA synthesis and the contraction of populations advancing through subsequent rounds of cell cycle.

In association with the increased DNA content of arrested LIG1-depleted cells, notable enlargement and altered adhesion properties were also observed (Supplementary Figures S1G, H). Whereas WT and *LIG3^−/−^:LIG4^−/−^* cells maintained an adherent and spindle-like morphology under 4-OHT treatment, the *LIG1^−/flox:CreERt2^* cells were strikingly transformed from self-aggregating rounded cells to flattened cells with increased topical adhesion and trypsin resistance when the second *LIG1* allele was deleted. This dramatic change in morphology in the absence of LIG1 was surprisingly even more prominent in cells pre-treated with nocodazole to induce a G2 cell cycle phase block (Supplementary Figure S1H). Nocodazole treatment was employed to potently arrest and enrich proportions of cells with ≥ 4N DNA content (Supplementary Figure S1F), recapitulating the effects of 4-OHT-induced *LIG1* deletion and permitting investigation of LIG1 function divorced from the influence of cell cycle progression. Importantly, nocodazole pre-treatment also stabilized the expression of LIG3 and LIG4 in response to 4-OHT, removing the potential for this variable to confound results (Figure 1C and Supplementary Figure S1B). As anticipated, WT, *LIG3^−/−^:LIG4^−/−^* and *LIG1^−/flox:CreERt2^* cells displayed enhanced mean cell diameters (captured in single cell suspension) under G2-arrest compared with their unsynchronized counterparts (WT; 1.21-fold, *P* = 0.002, *LIG3^−/−^:LIG4^−/−^*; 1.13-fold, *P* = 0.0003, *LIG1^−/flox:CreERt2^*; 1.10-fold, *P* = 0.0238) (Figure 1G). Even in the context of G2-arrest, the deletion of *LIG1* from *LIG1^−/flox:CreERt2^* cells resulted in a significant supplementary increase in cell size (1.11-fold; *P =* 0.0412) that equated to a greater than 1.5 micron difference in mean cell diameter. Thus, LIG1 depletion likely impacts cytoskeletal interactions in addition to DNA replication, resulting in stalled cell populations with replicated 4N DNA content and altered morphology and adhesion properties.

LIG1 depletion did not, however, result in any appreciable impairment of cell viability over longer term (7 days) culture (Supplementary Figure S1I). The patent alterations in cell morphology (Supplementary Figure S1I(i)) and size (1.5-fold greater in 4-OHT-treated compared with MeOH-treated; Supplementary Figure S1I(ii)) were exacerbated, whilst the depressed cell number (0.6-fold in 4-OHT-treated compared with MeOH-treated; Supplementary Figure S1I(ii)) underscores the cytostatic impact of the *LIG1* deletion evidenced in Supplementary Figure S1I(iii) and (iv). Interestingly, the reduced expression of senescence-associated genes, *DPP4* (55) and *HMGA2* (56), suggests that LIG1 depletion does not activate a global senescence program, whereas the moderate (1.2-fold) up-regulation of *CDKN1A* transcripts is indicative of increased DNA damage signalling in response to the ongoing replication stress (Supplementary Figure S1i(iv)). Hence, the morphological changes resulting from LIG1 depletion are likely associated with the onset of cellular quiescence (57), rather than senescence or apoptosis.

### LIG1 is absolutely required for sister chromatid telomere fusions

**Figure 2.**
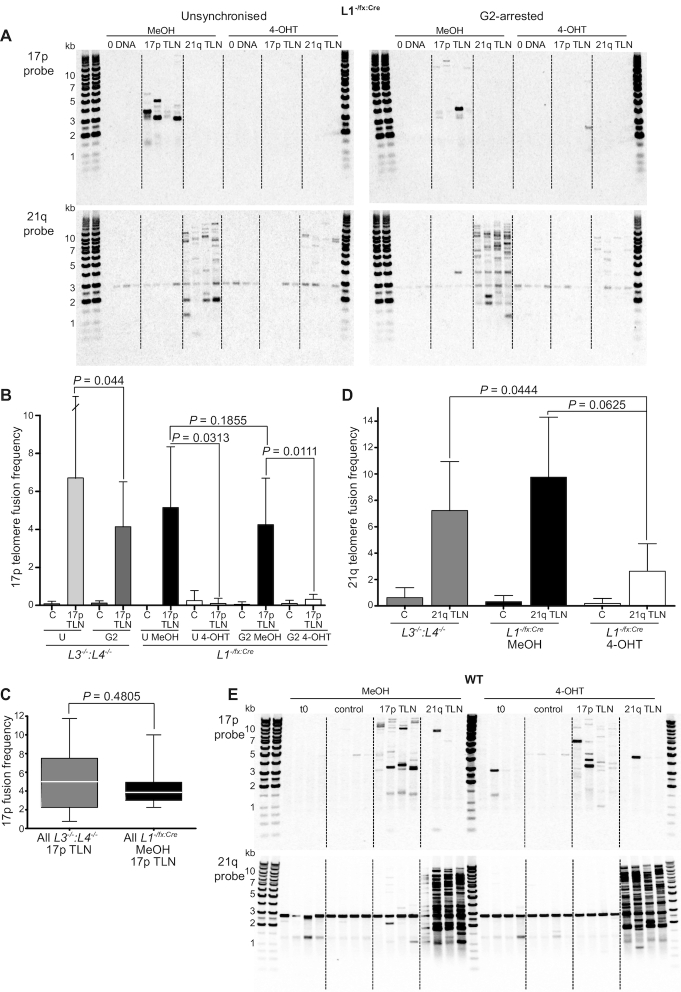
LIG1 is required for 17p sister chromatid telomere fusions. (**A**) Fusion amplicons derived from 17p or 21q TALEN (TLN) or control (0 DNA) -transfected unsynchronized or G2-arrested *L1^−/fx:Cre^* were visualized by Southern hybridization using probes specific for the 17p (upper panels) or 21q subtelomeric (lower panels) sequence. A 2.9 kb constitutive amplicon band of known sequence identity is also detected with the 21q probe, but excluded from all fusion amplicon analyses. (**B**) Summary of 17p telomere fusion frequency (fusions/ng DNA input x10^−2^) data from seven independent 17p TLN and control (**C**) transfection experiments in unsynchronized (U) and G2-arrested (G2) *L3^−/−^:L4^−/-^*and *L1^−/fx:Cre^* cells displayed as mean values with 95% confidence intervals with one-tailed paired T-tests or Mann–Whitney U-tests, as appropriate. (**C**) Box and whiskers representation of 17p telomere fusion frequency data presented in (b) as a pooled data comparison of all 17p TALEN-transfected *L3^−/−^:L4^−/−^* (grey) samples with all MeOH-treated *L1^−/fx:Cre^* (black) samples and compared by two-tailed Mann–Whitney U-test. (**D**) Summary of 21q telomere fusion frequency (fusions/ng DNA input ×10^−2^) data from 3 independent 21q TLN and control (C) transfection experiments in G2-arrested *L3^−/−^:L4^−/-^*and *L1^−/fx:Cre^* cells displayed as mean values with standard deviation and one-tailed paired T-tests or Mann–Whitney U-tests, as appropriate. (**E**) Southern hybridization images of G2-arrested WT 17p (upper panel) and 21q (lower panel) fusion amplicons generated as in (a). Untransfected (t0) and control (GFP) transfections are included for comparison with 17p and 21q TLN-transfected samples.

We have developed a model for studying telomere fusions uncoupled from the selective pressure of telomere attrition and crisis (21). To discriminate the relative roles of DNA ligases in telomere fusions in this study, we employed a similar strategy using transient transfection of Transcription Activator-like Effector Nucleases (TALEN) designed to cleave precise subtelomeric locations at the unique 17p telomere or the 16p/21q families of homologous chromosome ends that consists of at least 19 distinct telomeres (36). The 17p TALEN recognition site is immediately adjacent to the telomere repeat array, whereas the 21q TALEN targets sequences common to the 16p/21q telomere families located ∼1.5 kb from the start of the canonical telomere repeats. Since LIG1-depletion resulted in a clear and durable cell cycle arrest and accumulation of G2 phase cells (Figure 1E, F, Supplementary Figure S1D–F), the exploration of TALEN-induced telomere fusions was performed in both unsynchronized cells and those arrested in G2 by nocodazole pre-treatment to homogenize this effect across all cell lines compared. G2 conditions were perpetuated post TALEN transfection so that both damage induction and repair occurred within a single phase of the cell cycle when sister chromatids were available as fusion substrates and without the incremental constraints of cytokinesis. Telomere fusions were detected by Southern hybridization following single molecule long-range PCR (Figures 1H and 2A) using unidirectional 17p, XpYp and 21q family chromosome-specific primers (Supplementary Table S1) that yield amplicons only from covalently-linked fused or recombined chromosomes.

Transfection with the 17p TALEN robustly induced 17p telomere fusions in MeOH carrier control-treated *LIG1^−/flox:CreERt2^*cells under both unsynchronized and G2-arrested conditions, as detected with a 17p subtelomere- but not with a 21q subtelomere-specific hybridization probe (Figure 2A and a comparison with LIG1-expressing *LIG3^−/−^:LIG4^−/−^* cells shown in Supplementary Figure S2A). These fusions could be correspondingly amplified using a single 17p subtelomere primer, alone (Supplementary Figure S2B), consistent with originations in sister chromatid telomere fusion. In contrast to the MeOH carrier control-treated *LIG1^−/flox:CreERt2^*cells, the LIG1-depleted cells demonstrated a remarkable and invariable loss of 17p TALEN-induced sister chromatid fusions under all conditions tested (Figure 2A and Supplementary Figures S2A and B). In unsynchronized *LIG1^−/flox:CreERt2^* cells, 17p telomere fusion frequency was reduced more than 50-fold (*P* = 0.0313) by 4-OHT-mediated *LIG1* deletion (Figure 2B, central bars), complementing the potent cell cycle inhibition observed (Figure 1E, F, Supplementary Figure S1D–F). However, 17p fusions were still 13.2-fold lower in frequency (*P =* 0.0111) when LIG1 was depleted from cells already arrested in G2 phase (Figure 2B, far right bars), indicating additional cell cycle-independent consequences of the loss of this ligase. Whilst the transfection efficiency of LIG1-depleted cells was lower than for WT, *LIG3^−/−^:LIG4^−/−^* and carrier controls (Supplementary Figure S2C), adjusting fusion assay DNA input accordingly did not disclose latent fusions (Supplementary Figure S2D), so that the deficit is unconnected to assay efficiency at the limits of detection.

Importantly, these results also indicate a lack of functional compensation in telomere fusion end-joining by LIG3 and LIG4 expressed by *LIG1^−/flox:CreERt2^* cells (Figure 1C and Supplementary Figure S1I(iv)). Direct comparison of 17p fusion frequency in *LIG3^−/−^:LIG4^−/−^* cells that express only LIG1 (although maintaining expression of the essential mitochondrial-localized LIG3) (21) with *LIG1^−/flox:CreERt2^* cells sufficient for all three ligases revealed no substantial or statistically significant difference (*P* = 0.4805, Figure 2C and Supplementary Figure S2A), affirming that LIG1, alone, is sufficient, as well as necessary for sister chromatid fusion. Fusion frequency was comparable between LIG1-expressing unsynchronized and G2-arrested *LIG1^−/flox:CreERt2^* cells (*P* = 0.1855, Figure 2B), further underlining the more substantial impact of LIG1 expression than cell cycle status for the occurrence of 17p TALEN-induced telomere fusions. The minor, but consistent, 1.62-fold (*P* = 0.044, Figure 2B and Supplementary Figure S2A) reduction in 17p fusions in G2-arrested compared with unsynchronized *LIG3^−/−^:LIG4^−/−^* cells supports the hypothesis that LIG1 ligates TALEN-damaged sister chromatids juxtaposed following replication, so that fusion frequency is enhanced as a function of repeated passage of unsynchronized cells through G2 phase.

Whereas the 17p TALEN induces a single subtelomeric DSB initiating predominantly intra-chromosomal sister chromatid telomere fusions, the 21q TALEN induces DSBs in at least 19 distinct subtelomeres, so that the resulting telomere fusions can arise as a consequence of either inter- or intra-chromosomal recombinations. Variations in 21q TALEN-induced telomere fusion frequencies between LIG1-expressing and -depleted cells were notably more equivocal than those involving the 17p telomere (Figure 2A, D). A 2.76-fold reduction (*P* = 0.0444) in 21q fusions was measured for 4-OHT-treated *LIG1^−/flox:CreERt2^* cells expressing only LIG3 and LIG4 compared with G2-arrested *LIG3^−/−^:LIG4^−/−^* cells expressing only LIG1. Although not statistically-significant, a congruent 3.72-fold (*P* = 0.0625) depreciation in 21q fusion frequency was determined for *LIG1^−/flox:CreERt2^* cells following *LIG1* deletion. These observations are compatible with a suppression of sister chromatid fusions, but not inter-chromosomal fusions involving the related 16p and 21q telomeres in the absence of LIG1 (Figure 2D and lower 21q probe panels of Figure 2A and Supplementary Figure S2A). Thus, the more overt and persistent differential in 21q fusion frequency between the *LIG3^−/−^:LIG4^−/−^* and LIG1-depleted *LIG1^−/flox:CreERt2^* cells than between haploinsufficient MeOH or 4-OHT-treated *LIG1^−/flox:CreERt2^* logically correlates with *LIG1* allelic dosage and expression. However, the considerable homology shared amongst 16p/21q telomere family members obviates formal verification of the particular requirement for LIG1 in sister chromatid fusions since inter- and intra-chromosomal fusions cannot be readily distinguished for independent quantification. In stark contrast, WT cells replete for all ligases sustained a robust 17p and 21q TALEN-induced fusion response under conditions of both asynchrony (Supplementary Figure S2E) and G2-arrest following treatment with 4-OHT or carrier control (Figure 2E). Ergo, NHEJ-mediated repair per se, comprising a diversity of inter- and intra-chromosomal recombinations, is not cell cycle-restricted.

To cross-validate these observations, we employed a LIG1-specific chemical inhibitor, L82 (23,46) (provided by Dr Alan Tomkinson, University of New Mexico, USA) to examine whether this would correspondingly prohibit sister chromatid telomere fusions. Treatment of WT and *LIG3^−/−^:LIG4^−/−^* cells with even moderate levels (5 μM compared with *in vitro* IC_50_ of 12 ± 2 μM (23)) of this inhibitor resulted in augmented proportions of G2/M phase cells (defined by pHH3 expression) compared with untreated cells assayed at 12 and 24 h time points (Supplementary Figure S2F(i); central panels). Transient expansion of the G2/M fraction in response to L82 was described previously in accordance with the S-phase block caused by LIG1 functional inhibition (Supplementary Figure S2F(i)). Appropriately, L82 had the inverse effect in HCT116 *TP53^−/−^* cells (1.41-fold reduction; Supplementary Figure S2F(i) lower tier and summary plots) that have compromised cell cycle checkpoint regulation (58) and hence failed to arrest in response to replication errors elicited by LIG1 inhibition. Whilst the increase in mitotic fractions was comparable for WT and *LIG3^−/−^:LIG4^−/−^* cells after 12 h, the proliferative capacity of the *LIG3^−/−^:LIG4^−/−^* cells was notably subdued by L82 over 24 h, resulting in a reduced fold change compared with the WT (Supplementary Figure S2F(i), lower panel and summary charts). Hence, L82 has a more potent impact on cells expressing only LIG1. This is further illustrated in Supplementary Figure S2F(ii) that presents the 1.74-fold lesser increase in population growth for L82- compared with DMSO carrier control-treated *LIG3^−/−^:LIG4^−/−^* cells over 48 h (upper panel). This suppressed proliferation corresponded with a 1.67-fold contraction of S-phase cell fractions (central and lower panels), as expected from the inhibition of LIG1 catalytic activity that is required for DNA replication.

Vitally, *LIG3^−/−^:LIG4^−/−^* cells treated with cytostatic (50 μM) doses of L82 suffered a pronounced decrease in 17p TALEN-induced sister chromatid fusions amplified from both unsynchronized and G2-arrested cells (Supplementary Figure S2G(i), upper panels, G(ii), left panel) that paralleled the loss of 17p sister chromatid fusions associated with *LIG1* deletion in *LIG1^−/flox:CreERt2^* cells (Figure 2A and Supplementary Figure S2A). The suppression was, again, more marked under conditions of G2-arrest (2.35-fold reduction, *P* = 0.014), emphasising the considerable contribution of LIG1 to DNA repair in this phase. The deficit in 17p fusions was not accompanied by a proportionate reduction in 21q TALEN-induced fusions (Supplementary Figure S2G(i), lower panels, G(ii), right panel), in keeping with the lesser impact of *LIG1* deletion for these fusions that conceivably represent predominantly inter-chromosomal events. These results underscore the key and non-redundant contribution of LIG1 to sister chromatid but not uniquely to inter-chromosomal telomere fusions.

### LIG1-depleted cells display an intact DNA damage response

**Figure 3.**
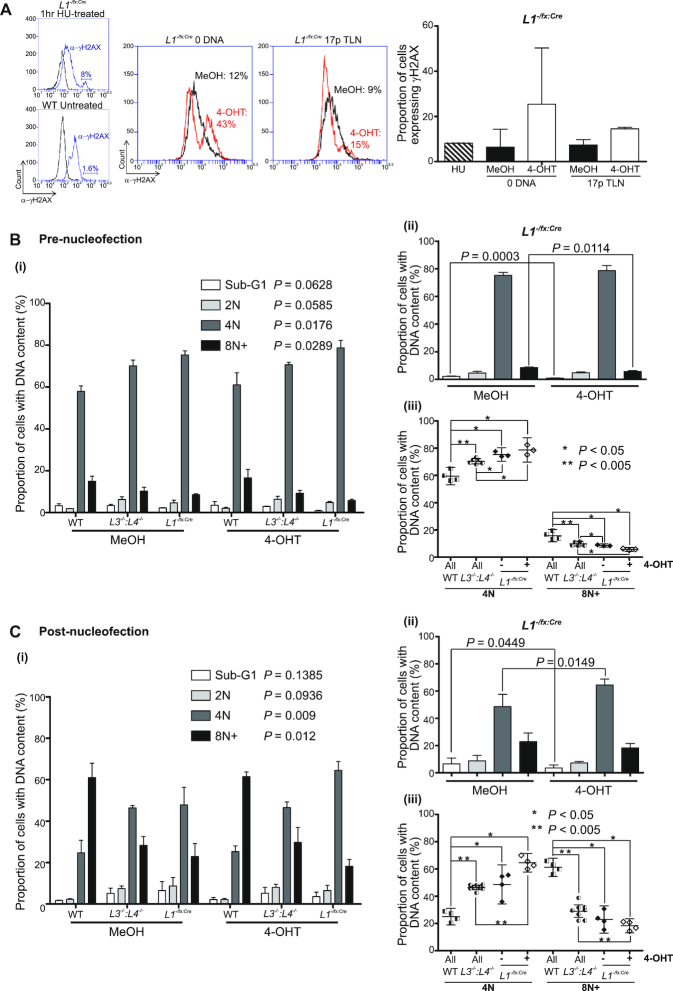
LIG1-depleted cells maintain an intact DNA damage response and suppressed mitotic slippage. (**A**) Flow cytometry detection of the phosphorylated (Ser139) histone variant H2AX (γH2AX; red overlays) as a surrogate marker for DSBs in G2-arrested and 4-OHT-treated *L1^−/fx:Cre^* cells transfected with 17p TLN or 0 DNA control over 24 h. The top left insert shows a histogram of γH2AX-expressing (8% population; blue overlay) unsynchronized and untreated *L1^−/fx:Cre^* cells exposed to 2 mM hydroxyurea (HU) for 1 h at 37°C. The bottom left insert reveals the basal 1.6% γH2AX-expressing asynchronous and untransfected WT HCT116 cells. The right-hand bar chart shows mean proportions (with SD) of γH2AX-expressing G2-arrested *L1^−/fx:Cre^* cells 24 h post-nucleofection (two replicas). The proportions of cells with defined DNA content in three independent experiments as assessed by DAPI DNA staining of fixed G2-arrested WT, *L3^−/−^:LIG4^−/−^* and *L1^−/fx:Cre^* cells (**B**) pre-nucleofection or (**C**) 24 h post-nucleofection (pooled 17p TLN and 0 DNA transfections) with detection by image cytometry. (i) Data for each fraction of pre- and post-nucleofection samples is plotted as the mean with standard deviation and assessed using one-way Kruskal–Wallis ANOVA of individual population fractions (significance set as alpha = 0.05) recorded against the colour key code. Differences relating specifically to the 4-OHT-mediated deletion of *LIG1* from *L1^−/fx:Cre^* cells are shown in B(ii) and C(ii). Significance was assessed by one-tailed paired T-tests of common fractions. The significant (Mann–Whitney U-tests, 0.05; *, 0.005; **) variations in proportions of WT, *L3^−/−^:L4^−/−^* and *L1^−/fx:Cre^* cells treated with 4-OHT are displayed in B(iii) and C(iii).

The conspicuous cell cycle-independent decreases in telomere fusion frequencies detected in LIG1-depleted HCT116 could result from attenuated DNA damage responses (DDR) to TALEN-induced DSB. To determine whether the source of the differential fusion frequency related to DNA damage sensing, rather than DNA repair activity, we measured phosphorylation of histone H2AX (γH2AX) as a biomarker of DSB (Figure 3A). Untransfected proliferating HCT116 cells routinely exhibit a baseline γH2AX activation in *circa* 1–2% of the total population (Figure 3A, WT Untreated histogram), however, G2-arrest and transfection elevated this baseline >10-fold to levels exceeding those measured in response to hydroxyurea-induced replication stress (8% γH2AX-staining cells; Figure 3A). Notably, 4-OHT-mediated *LIG1* deletion resulted in increased, rather than diminished populations of γH2AX-expressing cells in samples transfected with control DNA plasmids (Figure 3A, right panel), evidencing the clear capacity of *LIG1^−/flox:CreERt2^* cells for DDR activation following abrogation of DNA replication. Transfection with 17p TLN did not further exacerbate the detected DDR, conceivably owing to more extensive cell death in these samples. Whilst the differences in mean proportions of MeOH versus 4-OHT-treated γH2AX-expressing *LIG1^−/flox:CreERt2^* cells were not statistically significant, these data affirm that LIG1-depleted cells are competent to sense and respond to DNA damage, coordinate with the robust cell cycle arrest indicative of checkpoint activation (Figure 1E, Supplementary Figure S1D). Furthermore, we noted that arrest of LIG1-depleted cells in G1 or G2 cell cycle phases did not promote the onset of a senescent phenotype (as measured by expression of HMGA2 (56), DPP4 (55) (Supplementary Figures S1I(iv) and S3A) and IL6 (59) (undetected) that could confer resistance or impede responses to DSB (60,61).

### Mitotic slippage is suppressed in ligase-deficient cells

Prior to nucleofection, G2-arrested WT, *LIG3^−/−^:LIG4^−/−^* and *LIG1^−/flox:CreERt2^* samples displayed significantly divergent proportions of cells with defined DNA content (Figure 3B(i)). Although all samples were unambiguously enriched for populations with 4N (replicated) DNA content, proportions with this ploidy were significantly elevated in *LIG1^−/flox:CreERt2^* subsequently treated with or without 4-OHT (Figure 3B(i); *P* = 0.0176, B(ii), B(iii); *P* < 0.05). In contrast, the proportions of *LIG1^−/flox:CreERt2^* cells with ≥ 8N DNA content (representing polyploidy) were significantly lower than for WT and *LIG3^−/−^:LIG4^−/−^* cells (Figure 3B(i); *P* = 0.0289, B(ii), B(iii); *P* < 0.05). *LIG1* deletion resulted in moderate reductions in both SubG1 apoptotic cells (Figure 3B(ii); 2.57-fold, *P* = 0.0003) and ≥8N fractions (1.49-fold, *P* = 0.0114), suggesting a potent G2-arrest. The lower ≥8N fraction in LIG1-depleted cells than WT (2.76-fold *P* = 0.0286) *LIG3^−/−^:LIG4^−/−^* (1.71-fold *P* = 0.0119) and *LIG1^−/flox:CreERt2^* MeOH-treated (1.49-fold *P* = 0.0114) cells (Figure 3B(ii) and (iii)) indicates a suppression of mitotic slippage and endoreplication towards a tetraploid state that could be expected to provide additional target substrate for TALEN cleavage activity.

Post-nucleofection, the discrepancies between proportions of 4N and ≥8N (8N+) DNA content populations in WT, *LIG3^−/−^:LIG4^−/−^* and *LIG1^−/flox:CreERt2^* cells were further exaggerated (Figure 3C(i)). LIG1 depletion from the *LIG1^−/flox:CreERt2^* cells, again, resulted in a significant reduction in SubG1 constituents (1.83-fold, *P* = 0.0449), accompanied by an inflation of the 4N (1.33-fold, *P* = 0.0149), rather than the ≥8N populations (Figure 3C(ii)) and signifying a heightened G2-arrest following transfection. TALEN expression or activity had minimal impact on these ploidy distributions in *LIG1*-haploinsufficient or -deleted cells (Supplementary Figure S3B, *P* > 0.5). Substantial enlargements of the ≥8N distributions were observed for all samples (Figure 3C(i)), but the amplification was most striking amongst WT samples, for which this component rose from ∼16% to ∼61% of the total post-nucleofection and was 3.36-fold greater (*P* = 0.0143) than the equivalent fraction of LIG1-depleted cells (Figure 3C(iii)). *LIG3^−/−^:LIG4^−/−^* cells also demonstrated a greater proportion of ≥ 8N cells than the *LIG1^−/flox:CreERt2^* cells post-nucleofection that was further distended to a 1.59-fold difference (P = 0.002) with *LIG1* deletion (Figure 3C(iii)). The incrementally skewed ploidy of WT, *LIG3^−/−^:LIG4^−/−^* and *LIG1^−/flox:CreERt2^* cells echoes the frequencies of TALEN-induced telomere fusions detected in these samples, such that the WT cells exhibited the highest frequency of telomere fusion and largest proportions of 8N+ cells, whereas the *LIG1^−/flox:CreERt2^* cells displayed the lowest frequency of fusions and 8N+ cells (compare Figure 2A, E and Supplementary Figures S2A, E). These observations implicate polyploidy as an indicator of, or potential underlying mechanism contributing to, A-NHEJ-mediated telomere fusion and genomic instability (62). Incidence may, therefore, be a product of proliferative index in combination with capacity for DNA repair following transfection, resulting in differential prevalence and tolerance of mitotic slippage. However, the modest 1.49-fold (*P* = 0.0114) pre-nucleofection and 1.25-fold (*P* = 0.1808, NS) post-nucleofection reductions in ≥ 8N proportions in *LIG1^−/flox:CreERt2^* cells versus LIG1-depleted *LIG1^−/flox:CreERt2^* cells do not equate with the profound suppression of 17p sister chromatid fusions determined (Figure 2A, upper panel). Thus, the relative functions of LIG1 in DNA replication and repair contribute independently and potentially cumulatively to the incidence of telomere fusions.

### LIG1-mediated sister chromatid fusions amplified from G2 cells display asymmetric error rates

**Figure 4.**
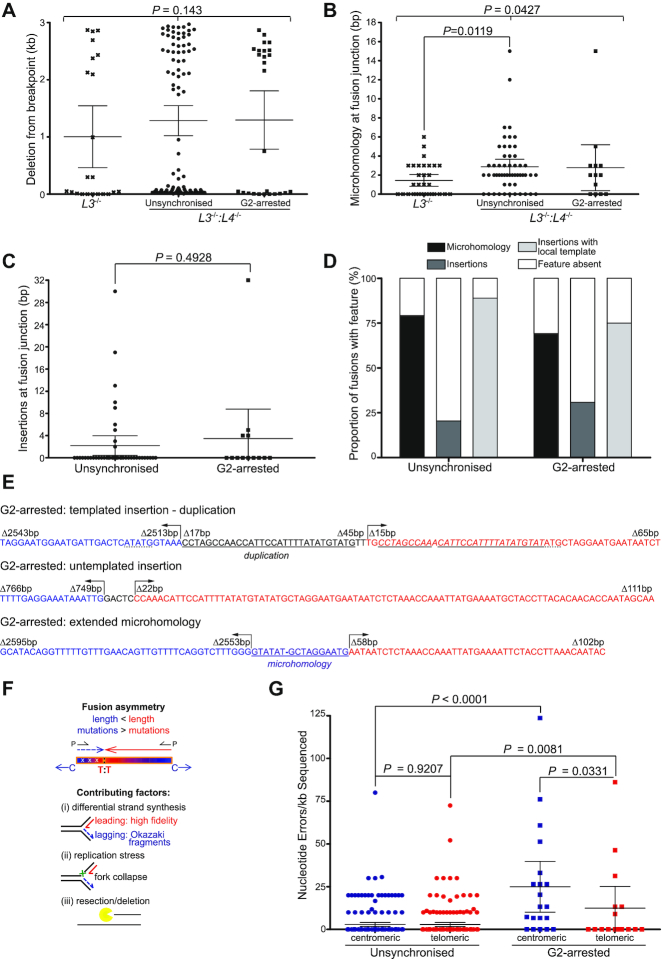
LIG1-mediated sister chromatid telomere fusions resolved by high-resolution fusion amplicon sequencing. Illumina paired-end sequencing of 17p telomere fusion amplicons derived from 17p TLN-transfected unsynchronized *L3^−/−^* and unsynchronized or G2-arrested *L3^−/−^:L4^−/−^* HCT116 was analysed for features of A-NHEJ-mediated repair. (**A**) The distance from the 17p TLN cleavage site to the fusion junction of each contributing chromatid within 17p intra-chromosomal fusions (*L3^−/−^:L4^−/−^*) or inter-chromosomal fusions (*L3^−/−^*) is plotted as mean deletion in kb with 95% confidence intervals and one-way Kruskal–Wallis ANOVA between samples. (**B**) The number of nucleotides (bp) with shared identity (microhomology at fusion junction) amongst participating sister chromatids constituting each 17p intra-chromosomal fusion (*L3^−/−^:L4^−/−^*) or across 17p inter-chromosomal (*L3*^−/−^) fusion junctions is presented with means and 95% confidence intervals, one-way Kruskal–Wallis ANOVA and two-tailed Mann–Whitney U-tests. (**C**) The number of nucleotides (bp) inserted within 17p intra-chromosomal *L3^−/−^:L4^−/-^*sample fusion junctions (not applicable to *L3*^−/-^ inter-chromosomal fusions) is shown with means and 95% confidence intervals with a two-tailed Mann–Whitney U-test. (**D**) The frequencies of events described in a-c are illustrated as a stacked column chart with colour key for unsynchronized and G2-arrested *L3^−/−^:L4^−/-^*samples. (**E**) Examples of 17p intra-chromosomal sister chromatid fusions mapped by Illumina paired-end sequencing from G2-arrested 17p TLN-transfected *L3^−/−^:L4^−/−^* cells. The centromeric (centromere-telomere orientation) component of each read pair is shown in blue, the telomeric (telomere-centromere orientation) component in red. Arrows indicate the direction of sequence and Δbp signifies the distance from the 17p TLN cleavage site. Templated (duplication) insertion and microhomology sequences underlined. (**F**) The centromeric components of sister chromatid fusion read pair (blue) have shorter mean lengths and increased mean nucleotide error incorporation rates than the telomeric components (red), resulting in asymmetric fusion molecules. Differences in (i) the fidelity of polymerase activity in leading (POLD/E) and lagging (POLA/POLD) strand DNA synthesis, as well as (ii) replication stress and fork collapse exposing lagging strand Okazaki fragments and ligation intermediates and (iii) strand bias in resection and/or deletion may contribute to both manifestations of asymmetry. C and T indicate the direction to the chromatid centromere and telomere, respectively. (**G**) Nucleotide incorporation error rate was calculated for both the centromeric (blue) and the telomeric (red) components of each 17p sister chromatid fusion read pair sequenced from *L3^−/−^:L4^−/−^* cells and presented with means and 95% confidence intervals. One-way Kruskal–Wallis ANOVA and two- (unsynchronized) or one (G2-arrested) -tailed Mann–Whitney U-tests were employed.

Our previous high-resolution characterization of telomere fusions mapped from Illumina paired-end sequencing yielded valuable insights into the molecular features of specific DNA repair activity (21). To further examine the consequences of LIG1-mediated sister chromatid telomere fusion, we focussed on G2 cell cycle phase cells in which LIG1 DNA repair activity could be functionally uncoupled from its requisite role in DNA replication. We performed Illumina paired-end sequencing of pooled 17p-17p (single primer) telomere fusion amplicons derived from multiple transfections of G2-arrested *LIG3^−/−^:LIG4^−/−^* cells with 17p TALEN nucleases harvested after 24 h. Discordant read-pairs aligned in an orientation predicted to arise as a consequence of sister chromatid 17p fusion were identified and sequence-validated to define precise fusion junctions. This dataset was juxtaposed with the 48 h subset extracted from our former pooled 48 and 120 h Illumina sequencing datasets of 17p TALEN-induced sister chromatid fusions amplified from unsynchronized *LIG3^−/−^:LIG4^−/−^* cells (21) in novel analyses intended to spotlight LIG1-specific repair features (Figure 4). Since LIG4 is essential for inter-chromosomal and not intra-chromosomal fusions, we performed comparisons of the *LIG3^−/−^:LIG4^−/−^* datasets with only inter-chromosomal telomere fusions derived from a nuclear *LIG3*^−/−^ HCT116 model of LIG4-dependent C-NHEJ described in the same study (21).

As anticipated, telomere fusions were characterized by a stereotypical length asymmetry of contributing sister chromatids that resulted in bi-modal distributions of deletion distances from the TALEN-cleaved breakpoint (Figure 4A). These deletion profiles were analogous (*P* = 0.143) amongst both *LIG3^−/−^:LIG4^−/−^* datasets (unsynchronized mean of 1.28 kb, G2-arrested mean of 1.3 kb) and only moderately divergent from the *LIG3*^−/−^-derived fusions (mean of 1.0 kb). There was a comparable parity of microhomology usage (Figure 4B) at fusion junctions of unsynchronized (mean of 2.87 bp) and G2-arrested (mean of 2.77 bp) *LIG3^−/−^:LIG4^−/−^* cells, suggesting a commonality of underlying processes, regardless of compromised cytokinesis and replication stalling in G2-arrested cells. Microhomology usage at *LIG3*^−/−^ inter-chromosomal fusion junctions was prominently 2-fold lower than for the *LIG3^−/−^:LIG4^−/−^* cells (*P* = 0.0427), affirming a more error-prone mechanism of DSB DNA repair at subtelomeres prosecuted by LIG1 and consistent with former definitions of A-NHEJ (21,35,36). Small (<50 bp) sequence insertions were detected at fusion junctions resolved from both unsynchronized and G2-arrested *LIG3^−/−^:LIG4^−/−^* cells (Figure 4C–E) with corresponding *(P* = 0.4928) distributions (unsynchronized mean of 2.21 bp, G2-arrested mean of 3.46 bp). Comparison with C-NHEJ-mediated (*LIG3*^−/−^) inter-chromosomal fusions was not appropriate since these events incorporate diverse genomic insertions. Rare fusions with long (>10 bp) stretches of microhomology or larger (>15 bp) insertions at the fusion junctions were evident in both *LIG3^−/−^:LIG4^−/−^* samples at indistinguishable levels. Insertions were predominantly templated from adjacent sequence regions (Figure 4D–E), emblematic of DNA polymerase theta (POLQ) engagement (63,64). These results serve to reinforce the supposition that LIG1 is competent and sufficient to effect A-NHEJ at telomeres, resulting in fusion of asymmetric sister chromatids via notable microhomology usage and POLQ-mediated DNA synthesis.

To further explore the asymmetry of fused sister chromatids, we compared the sequence of centromeric (centromere-telomere orientation of single fusion amplicon sequenced 5′-3′; more extensively-deleted) and telomeric (telomere-centromere orientation; minimally-deleted) constituents of each fusion amplicon read-pair (illustrated in Figure 4F). Rationally, we identified an elevated error rate per kilobase (kb) for all sequence read-pairs derived from G2-arrested compared with unsynchronized *LIG3^−/−^:LIG4^−/−^* cell samples (Figure 4G). Specifically, the centromeric (blue symbols) read-pair components sequenced from G2-arrested cells displayed an 8.55-fold higher error rate than centromeric read-pair components from unsynchronized cells (*P* < 0.0001) and a correspondent 4.32-fold (*P* = 0.0081) increase in errors within telomeric read-pair components was also identified. These observations may evince the particular activity of LIG1 localized at stalled replication forks within late-replicating subtelomeric heterochromatin that subsequently mature into telomere fusions under prolonged G2-arrest (Figure 4F). More intriguingly, the relative sequence fidelity of centromeric and telomeric fusion read-pair components also differed significantly within the G2-arrested *LIG3^−/−^:LIG4^−/−^* sample (Figure 4G). The rate of erroneous nucleotide incorporation into the shorter centromeric read-pair components (blue symbols) was double (*P* = 0.0331) that detected for the telomeric read-pairs (red symbols). This differential repair fidelity denotes an asymmetry of mutational load for the fused sister chromatids that complements the length asymmetry described in Figure 4A and portrayed in Figure 4F. Polarized length and mutational burden of contributing sister chromatids may reflect the intrinsic inequality of strand synthesis and LIG1-dependent Okazaki fragment ligation (Figure 4F).

### Ligase-depleted cells maintain residual capacity for inter-chromosomal telomere fusion

Whereas LIG1-expressing *LIG3^−/−^:LIG4^−/−^* cells were replete for 17p sister chromatid and 16p/21q family inter-chromosomal telomere fusions (Supplementary Figure S2A), the sole deletion of *LIG1* had a palpably more prohibitive impact on sister chromatid fusions than on inter-chromosomal fusions (Figure 2A–D, Supplementary Figure S2A). These observations raise the possibility of ligase-independent processes, as well as complementary involvement of LIG1 and LIG4, in these long-range interactions in G2-arrested cells. To discern ligase-independence or redundancy, we generated and employed additional HCT116 genetic models. Two haploinsufficient *LIG1* conditional deletion clones (C15-5 and C15-9) were created from the *LIG3^−/−^:LIG4^−/−^* HCT116 cell line (Supplementary Figures S4A–C), so that 4-OHT could, again, activate Cre-mediated excision of the single remaining *LIG1* allele to rapidly render cells devoid of all nuclear DNA ligases under asynchronous (Supplementary Figure S4C) or G2 cell cycle conditions (Supplementary Figure S4D). Accordingly, *LIG1* deletion was associated with a prominent 1.5-fold decrease in LIG1 protein (Supplementary Figure S1C) corresponding to a 1.5-fold decrement (*P* = 0.0096) in LIG1 mRNA measured after 24 h (Supplementary Figure S4C(iii)). Consistent with our observations with the *LIG1^−/flox:CreERt2^* cells (Supplementary Figure S1G, H), 4-OHT-mediated LIG1 depletion reproducibly induced considerable alterations in the morphology of the *LIG3^−/−^:LIG4^−/−^:LIG1*^−/flox:CreERt2^ clones (Supplementary Figure S4D(ii)), further evidenced by the significant ∼ 1.1-fold increases in mean cell diameter (Supplementary Figure S4D(iii), unsynchronized C15-5; *P* = 0.0019, C15-9; *P* = 0.0047, G2-arrested C15-5; *P* = 0.0172, C15-9; *P* = NS). These data reinforce our suppositions that LIG1 function has diverse effects on subcellular organization that may be direct or indirect via DNA damage signalling and cell cycle checkpoint activation.

**Figure 5.**
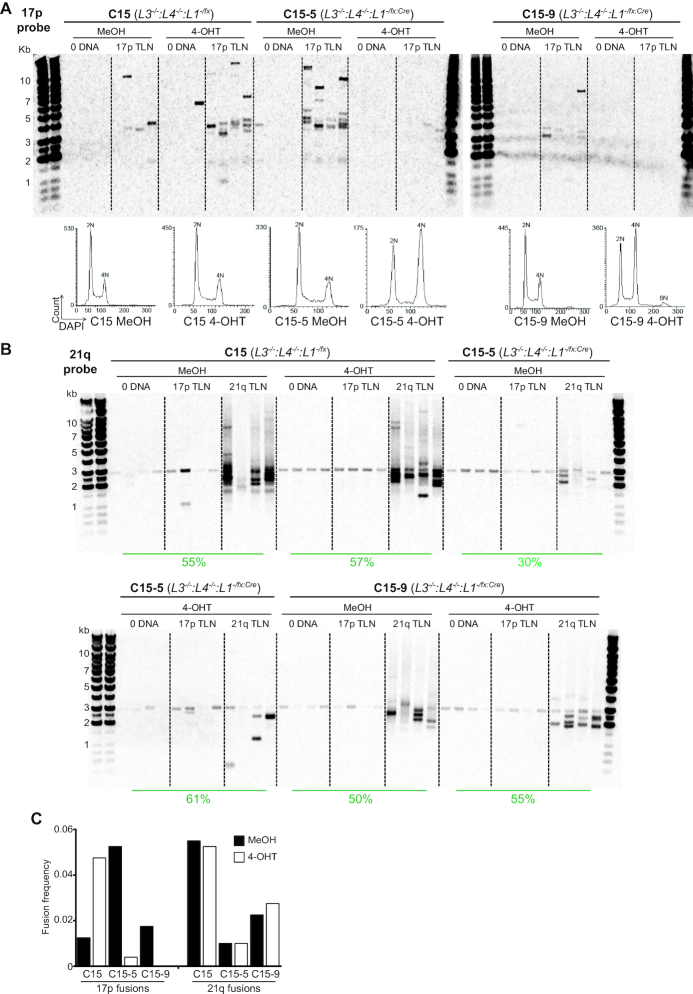
Ligase-depleted cells retain inter-chromosomal fusion capacity. (**A**) Southern hybridization detection of 17p fusions amplified from control (0 DNA) and 17p TLN-transfected unsynchronized and 4-OHT-treated *L3^−/−^:L4^−/−^L1^−/fx:Cre^* (clones C15-5 and C15-9) and parental clone C15 (*L3^−/−^:L4^−/−^L1^−fx^*) cells harvested after 48 h. Corresponding DNA content for each sample at the point of transfection is displayed below as linear histograms with annotated ploidy peaks. (**B**) Southern hybridization detection of 21q fusions induced by transfection of G2-arrested and 4-OHT-treated *L3^−/−^:L4^−/−^L1^−/fx:Cre^* and *L3^−/−^:L4^−/−^L1^−fx^* cells with 17p and 21q TLN over 48 h. Transfection efficiency (%) of each sample at 24 h post-nucleofection is indicated in below the lanes. (**C**) Bar chart summary of 17p (left side) and 21q (right side) fusion frequency data displayed in (A) and (B) for parental *L3^−/−^:L4^−/−^L1^−fx^* clone C15 and *L3^−/−^:L4^−/−^L1^−/fx:Cre^* clones C15-5 and C15-9 in the presence of MeOH or 4-OHT.

Importantly, and comparable with the *LIG3*- and *LIG4*-proficient LIG1-depleted cells, 17p TALEN-induced sister chromatid fusions were abolished by 4-OHT treatment of the *LIG3^−/−^:LIG4^−/−^:LIG1^−/flox:CreERt2^* cells (Clones C15-5 and C15-9), but not parental clone lacking the CreERt2 transgene (C15) (Figure 5A). This loss of 17p sister chromatid fusions was coincident with cell cycle arrest and expanded proportions of ≥ 4N DNA content cells following 4-OHT-induced *LIG1* deletion (Figure 5A, DNA content histograms displayed in lower panel). In contrast, 21q TALEN-induced fusions persisted in LIG1-depleted *LIG3^−/−^:LIG4^−/−^:LIG1^−/flox:CreERt2^* cells even when standardising and restricting cell cycle progression using nocodazole (Figure 5B). Both *LIG3^−/−^:LIG4^−/−^:LIG1^−/flox:CreERt2^* clones showed reduced (5-fold; C15-5, 2-fold; C15-9) 21q fusion frequency compared with the parental line, C15 (Figure 5C), but LIG1 depletion in response to 4-OHT treatment did not further dramatically curtail these events. Transfection efficiencies of *LIG3^−/−^:LIG4^−/−^:LIG1^−/flox:CreERt2^* clones and parental samples were approximately equivalent and did not account for variations in fusion frequencies (Figure 5B, recorded below panels). Thus, a subset of telomere fusions activated by a subtelomeric DSB appear to arise from ligase-independent DNA repair mechanisms operating in post-replicative G2-arrested cells.

Interestingly, the 21q telomere fusions residually occurring in ligase-deficient cells demonstrated a contracted molecular weight distribution focussed within a 2–4 kb range and a notable absence of larger 8–12 kb amplicons, indicating alternative processing explicitly utilized in this repair pathway. These 21q telomere fusions were intractable to comprehensive sequence characterization, suggesting challenging structure or repetitive composition. Rare events that could be resolved revealed recombinations involving subtelomeric fragmentation and inversion (Supplementary Figure S5), conceivably diagnostic of repair processes such as single-strand annealing (65). Since the 21q TALEN pairs cleave multiple subtelomeres, fusions may incorporate diverse genomic loci or employ uncut homologous template for recombination or synthesis-based repair (66). Novel approaches to characterize these challenging templates will be required to fully understand the range of DNA repair options that can be substituted to safeguard genome stability.

## DISCUSSION

We previously postulated a role for LIG1 in mediating intra-chromosomal telomere fusions in the context of impaired A- and C-NHEJ resulting from the genetic ablation of nuclear LIG3 and LIG4, respectively. Our current data, derived from a novel conditional targeting of human *LIG1*, not only supports this hypothesis, but also reveals an absolute and non-redundant requirement for LIG1 in the fusion of nuclease-targeted sister chromatid telomeres post-replication.

### LIG1 depletion impairs replication in a rapidly-dividing human cancer cell line

Investigations into the particular contributions of LIG1 to DNA replication and repair have historically returned diverse and often contradictory results concerning the cellular requirements for and functions of this ligase. Targeted deletion of *LIG1* in mouse embryos was found to be lethal (67) and antisense oligonucleotides targeting *LIG1* transcripts in human tumours have achieved effective suppression of tumour growth (10). Conversely, other mammalian models (predominantly mouse) demonstrate a non-essential role for LIG1 (24–28,33,68,69) and propound functional substitution with LIG3. Discrepancies may relate to both the mechanisms and degrees of LIG1 elimination, as well as the experimental time-scale and specific processes investigated. Inherent differences between model organisms may also provide a heterogeneous context within which to examine LIG1 function, given the varied degrees of conservation of DNA ligases amongst eukaryotes (1). For these reasons, we have examined the unequivocal contribution of LIG1 to a specific DNA repair mechanism that operates at dysfunctional telomeres in a human cancer cell line. Our unique model allows for comparison of telomere recombination profiles amongst related cell lines with distinct repair pathway component deletions, thus permitting stringent resolution of key protagonists (21). The precise temporal control over LIG1 depletion afforded by our conditional recombination approach has permitted evaluation of the acute and direct impact of this ligase in specific repair processes at defined cell cycle stages.

During the development of our novel LIG1-deficient HCT116 cell line, we found homozygous null cells could never be propagated. This is consistent with an indispensable role of LIG1 in DNA replication, further underscored by the robust cell cycle arrest achieved within 24 h of the induced recombination of the remaining intact *LIG1* allele in *LIG1^−/flox:CreERt2^* cells. In addition, we noted the restricted supernumerary ploidy expansions of LIG1-depleted cells in response to DNA damage and replication stress. Notably, the transcription of *LIG3* and *LIG4* was moderately increased by the depletion of LIG1 in unsynchronized and G1 phase cells and largely unaffected in G2 phase cells, so these results corroborate an incapacity of LIG3 to compensate for the loss of LIG1 in supporting DNA replication. Hence, although the catalytic capabilities of LIG1 and LIG3 may be analogous (1), temporal, spatial or kinetic constraints (31,32) seemingly prevent functional complementation in rapidly-propagating cells. The translational corollary of this is that LIG1 may be a valuable target for cancer therapeutics since proliferating rather than normal healthy somatic cells would be more severely afflicted.

Our model also emphasizes the capacity of critical cellular processes to be maintained by minimal levels of key components. We determined no limitations in population growth rates or viability of heterozygous *LIG1* cells over short experimental time periods. In keeping with the survival of a patient with compound null and hypomorphic mutations in *LIG1* into her late teens (11,12) and the successful propagation of 46BR fibroblast lines derived from this same patient (14,15), attenuated LIG1 expression or activity does not abrogate cell viability. Furthermore, nuclease-induced telomere fusions were unequivocally amplified from *LIG1^−/flox:CreERt2^* and *LIG3^−/−^:LIG4^−/−^:LIG1:^−/flox:CreERt2^*cells harbouring only a single intact *LIG1* allele. Hence, although thresholds may determine repair outcomes and cell fate in certain circumstances (70), cellular dependence on LIG1 appears to be elemental rather than graduated, so that even diminished functional levels can effectively sustain DNA replication and repair.

### LIG1 depletion alters morphology and adhesion of cancer cells

The genetic ablation of *LIG1* exons encoding the catalytic and DNA binding domains rapidly instigated a remarkable transformation in cell morphology. We observed an increase in mean cell size that surpassed the customary enlargement associated with mitotic progression (71) and was unparalleled in the WT and *LIG3^−/−^:LIG4^−/−^* lines. This cellular expansion may reflect karyotypic and metabolic changes resulting from the impaired DNA replication and repair activity specifically in the LIG1-depleted cells. Increased cell volume may also be a factor of both micronuclei formation (29) and mitochondrial compensatory biogenesis in response to the replication stress caused by defective lagging strand synthesis (72,73) in LIG1-deficient cells. LIG1 insufficiency has also been associated with cellular ‘adaptation’ that describes ongoing cell division despite unresolved DNA lesions (54,74). Although we also determined substantial activation of DNA damage markers following *LIG1* deletion, our model employing nocodazole-induced G2-arrest and observations of reduced proportions of LIG1-depleted cells with ≥ 8N DNA content precludes explanations of mitotic slippage and polyploidy as agents of cellular expansion.

Instead, our data support the premise that LIG1 may impact genome integrity via modulation of cytoskeletal interactions and dynamics following the onset of quiescence (57). We detected considerable changes in adhesion properties of LIG1-depleted HCT116 that are in agreement with previous reports of differential cadherin expression in 46BR.1G1 LIG1-deficient fibroblasts (54,75). The dramatic transformation of rounded self-aggregating *LIG1^−/flox:CreERt2^* cells into flattened monolayers following 4-OHT treatment is indicative of substantial cytoskeletal reorganization with LIG1 depletion. Where previously cell cycle progression has been considered to be regulated by altered cell-cell and cell-matrix interactions (76), the collective descriptions of modified adhesion in LIG1-deficient cells raises the intriguing possibility that DNA replication and repair may coordinate the cytoskeletal rearrangements that precipitate mitotic rounding and cytokinesis (71,77). Changes in cellular adhesion impact tumour-stroma interactions, conferring resistance to DNA damaging therapeutics and predisposing to metastatic transformations (78–81). Thus, LIG1 provides scope to understand the fundamental and pervasive connections between DNA replication, repair, cytoskeletal organization and intracellular signalling that are transformative in cancer.

### LIG1 is necessary and sufficient for sister chromatid telomere fusion in the G2 cell cycle phase

We previously proposed a role for LIG1 in mediating telomere fusions in the absence of LIG3 and LIG4 (21). In the current study, we directly compared the incidence of TALEN-induced telomere fusions in LIG1-depleted HCT116 cells with those derived from cells expressing only LIG1 (*LIG3^−/−^:LIG4^−/−^*) and cells depleted of all three DNA ligases. We demonstrated the persistent and compelling occurrence of sister chromatid (17p) and inter-chromosomal (21q) telomere fusions in *LIG3^−/−^:LIG4^−/−^* cells under conditions of asynchrony and G2-arrest, establishing that LIG1, alone, is sufficient to support telomere recombinations comparable to ligase-replete WT cells. High-resolution sequencing of LIG1-mediated telomere fusions derived from *LIG3^−/−^:LIG4^−/−^* cells revealed features characteristic of the A-NHEJ repair implicated in fusions resulting from both telomere attrition (36) and nuclease-targeting (21), including microhomology usage and deletion at junctions. Notably, we identified fusions that contained insertions templated from adjacent sister chromatid sequence reminiscent of the templated insertions introduced during DNA POLQ-mediated end-joining (63,82,83).

In flagrant opposition, the singular deletion of *LIG1* from *LIG1^−/flox:CreERt2^* cells resulted in the complete abrogation of sister chromatid telomere fusions in G2-arrested cells that retained expression of LIG3 and LIG4. Whilst we were unable to formally prove that the 21q fusions sustained in the absence of LIG1 comprised inter-chromosomal rather than sister-chromatid fusions, the ∼3-fold decreases in the frequency of these events amplified from LIG1-depleted cells approximated the ∼13-fold reductions determined specifically for 17p sister chromatid fusions, suggesting the loss of a related subset of events. These results reveal a non-redundant requirement for LIG1 in distinct post-replicative DNA repair processes, as well as in replication per se. As for DNA replication, LIG3 and LIG4 do not provide functional compensation in sister chromatid telomere fusion, thus conflicting with the premise that ligases are operatively interchangeable (24,68). Importantly, though, our data are compatible with the failure of LIG3 to rescue the specific telomere instability induced by the shRNA-targeted depletion of LIG1 reported by Le Chalony *et al.* (26), as well as the unique capacity we described for LIG3 in facilitating escape from a telomere-driven crisis (34). Crucially, the remarkable requirement for LIG1 to mediate sister chromatid fusion in cells fully proficient for C-NHEJ negates the supposition that this constitutes a mere auxiliary repair pathway.

Post-replicative repair is critical both in quiescent cells (84) and in proliferating cells for the resolution of lesions that elicit replication fork stalling, compromising the completion of late-replicating loci including telomeres and other heterochromatin regions (85,86). Telomeres present a unique susceptibility to DNA damage following replication stress and fork collapse since their distal chromosomal locations precludes rescue by obverse fork movement. Structural complexities arising from G quadruplex and R-loop formation generate further obstacles to the replication machinery at telomeres, contributing to exacerbated replication stress and fork destabilization through the formation of RNA-DNA hybrids that expedite genomic instability (87,88). Hence, post-replicative telomere DNA repair must be regulated to restrict gross telomere deletion and recombination that could be lethal or transformative (89,90).

Mechanistically, LIG1 is optimally-positioned to coordinate DNA repair at the replication fork in S/G2 cell cycle phases (7) when it is juxtaposed with nascent lagging strand DNA (91). This uniquely capacitates LIG1 for the repair of TALEN-induced DSB in late-replicating subtelomeric sequence, elucidating the absolute requirement we uncovered for this ligase for sister chromatid telomere fusion. Our proposition that LIG1 profoundly influences cytoskeletal interactions may account, in part, for the categorical loss of these fusions when *LIG1* is deleted. Cytoskeletal dynamics are increasingly being recognized as integral to the transmission of the DDR (92) and translation into DNA repair pathway selection (93,94). Conceptually, the absence of LIG1 could affect subnuclear localization of damaged subtelomeres that, in the context of sustained replication stress and cell cycle arrest, could license more effective activation of homologous or heterologous recombination (66,95) in preference to NHEJ-mediated telomere fusion. Cytoskeletal dynamics are also of fundamental importance to chromosome motility and scheduled segregation at mitosis (77), suggesting an additional level of interconnection between LIG1 function and ploidy or aneuploidy. We documented a significant suppression of ploidy accretion in LIG1-depleted cells that could be expected to concomitantly reduce the opportunity for telomere fusion and constrain the consequent precipitous genomic instability (62,96,97). Recent elucidation of the mechanisms integrating DNA replication and sister chromatid cohesion (98) present additional possible explanations for the dearth of sister chromatid fusions in LIG1-deficient cells. As with other replication factors (99), LIG1 may be integral to coordinated replication and sister chromatid cohesion, such that the cohesion fatigue likely experienced by cells held in prolonged G2-arrest is heightened in its absence. Weaker or more transient cohesion could hence reduce interaction and, as a corollary, fusion incidence between damaged sister chromatid telomeres subjected to NHEJ. By association, impaired cohesion also facilitates more extensive DNA resection that assists homology-directed repair rather than NHEJ (98,100).

### Differential requirement for LIG1 at inter-chromosomal telomere fusions

Our data reveal a divergent dependence of sister and inter-chromosomal telomere fusions on LIG1 activity that highlights the co-existence of distinct and conceivably competitive DNA repair processes in post-replicative cells. Whereas 17p sister chromatid telomere fusions were undetectable in the absence of LIG1, 21q inter-chromosomal fusions between related homologous family members were less severely diminished. The LIG1 non-competitive inhibitor, L82 (23,46), also had limited effect on these inter-chromosomal fusions, although highly-effective in suppressing LIG1-dependent sister chromatid fusions. Thus, inter-chromosomal telomere fusions are predominantly mediated by repair that operates synonymously with or independently of LIG1 catalytic activity. Since the 21q TALEN cleaves multiple homologous chromosome ends, the disparity in LIG1 involvement may arize as a factor of lesion prevalence, as well as the engagement of explicit DNA repair. Feasibly, the augmented propensity for aneuploidy and mis-segregation intrinsic to chromosomes targeted by the 21q TALEN compared with the 17p TALEN may contribute to the activation of more global genomic instability, as well as a broader range of DNA repair mechanisms (101). Notably, interactions between damaged subtelomeres of different chromosomes will not share the same susceptibility to replication factor engagement as those consequent of sister chromatid cohesion (99), elucidating the differential impact of LIG1-depletion for these inter-chromosomal fusions. The contribution of LIG4 to such long-range inter-chromosomal recombinations has been established by our group and others, however, our current study reveals this function may be transposable by LIG1 and possible supplementary mechanisms in G2-arrested cells.

Intriguingly, an unanticipated residual capacity for inter-chromosomal telomere fusions was uncovered using cells deficient in all three DNA ligases. Whilst we cannot absolutely exclude the prospect that these fusions were prosecuted by residual or N-terminal truncated LIG1 proteins in the 4-OHT-treated *LIG3^−/−^:LIG4^−/−^:LIG1^−/flox:CreERt2^* cells, we detected no symptomatic recovery from the potent cell cycle arrest induced by *LIG1* deletion. Similarly, the potential for mitochondrial LIG3 to rescue the defective NHEJ in nuclear ligase-depleted cells endures, but has not formerly proven significant even in cells facing the severe selection pressures of a telomere-driven crisis (34). Importantly, the molecular weight distributions of telomere fusions amplified from *LIG1*-deleted *LIG3^−/−^:LIG4^−/−^:LIG1^−/flox:CreERt2^* cells were patently distinct from those amplified from LIG1-expressing (A-NHEJ-competent) cells. This diversification may implicate alternative ligase-independent repair mechanisms, including heterologous recombination (66), break-induced replication (102) or single-strand annealing. Further specialized investigation will be required to formally substantiate the ligase-independence and particular characteristics of these events, however.

### LIG1-mediated sister chromatid telomeric fusions display asymmetric processing

We determined recurrent asymmetry of deletion from the subtelomeric nuclease cleavage site of paired chromatids comprising each sister chromatid telomere fusion amplified from both unsynchronized and G2-arrested *LIG3^−/−^:LIG4^−/−^* cells. The close correspondence with preceding datasets derived from diverse DNA repair deficient models (21) intimates a common and fundamental involvement of LIG1 in these particular recombinations. Integration of the biochemical pathways in which LIG1 is involved provides a rationale for the length asymmetry of fused chromatids (illustrated in Figure 4F). Replication fork stalling in late-replicating subtelomeric sequence has the potential to result in the incomplete replication or truncation of one chromatid (103). LIG1 complexed with PCNA and unligated Okazaki fragments may nucleate fusion of leading and lagging strand DNA, facilitated by POLQ helicase activity (82,104) and stabilized by POLQ-mediated DNA synthesis (63,105). The templated insertions we observed at sister chromatid fusion junctions could equally arise as a consequence of the incorporation of ligation intermediates released by strand displacement DNA synthesis (31). The 17p sister chromatid telomere fusions we sequence-verified were predominantly composed of one chromatid truncated to the approximate length of an Okazaki fragment (<500 bp) and the other minimally-deleted from the TALEN cleavage site, compatible with a hypothesis of fusion as a recourse from or by-product of replication fork dysfunction. However, it remains plausible that the length asymmetry measured arises purely from disproportionate strand resection (106), generating substrates of discordant length for NHEJ.

Superimposed upon the length asymmetry, we observed a remarkable discrepancy in mutational load for the paired constituents of sister chromatid fusions amplified from *LIG3^−/−^:LIG4^−/−^* G2-arrested cells. The significantly higher rate of erroneous nucleotide insertion we detected for the shorter ‘centromeric’ than the longer ‘telomeric’ components of these fusion pairs necessarily relates to inherent differences in the synthesis or processing of these chromatids that subsequently fuse within the same cell. We have postulated a correlation of nucleotide error rate with the distinct DNA polymerases responsible for synthesising leading versus lagging DNA strands (21,107,108). As an extension of this, an asymmetry of mismatch repair focused at Okazaki fragments of the lagging strand has recently been reported (109) that could underpin the discrepant mutation frequency at fused sister chromatids comprising paired long ‘telomeric’ leading strand and short ‘centromeric’ lagging strand DNA. The apparent bias uncovered by the resolution specifically of G2 phase LIG1-mediated fusions may reflect a compounding decline in the repair of nucleotide damage incurred in exposed single-strand DNA (110) resulting from extensive resection or replication fork collapse in arrested cells. LIG1 is also a key component of the base excision repair that performs critical post-replicative processing of telomeric lesions to license faithful chromosome segregation at mitosis (111,112). Competing requirements for LIG1 engagement in the context of DNA damage combined with replication stress may contribute to the exacerbated and polarized mutation distinguished for sister chromatid fusions derived from G2-arrested cells.

## CONCLUDING REMARKS

We have demonstrated a unique, cell cycle-independent and non-redundant role for LIG1 in sister chromatid telomere fusions, thus securely establishing LIG1 as not only an essential engineer of mitosis, but also as a critical post-replicative repair enzyme. Central to both DNA replication and repair, LIG1 is uniquely-competent to orchestrate a coordinated response to lesions in late-replicating DNA, including at sites of replication fork collapse and telomeric sequences. As such, LIG1 may constitute a final opportunity to resolve persistent damage ahead of the mitotic progression that would expedite global and catastrophic genomic instability. We therefore propose LIG1 as an attractive therapeutic target for both oncology, where accelerated proliferation jeopardises consonant DNA synthesis and repair, as well as pathological contexts where the quality of post-replicative repair may critically influence disease progression. Importantly, our data provide further clarification for the separation of function of DNA ligases within mammalian cells that will be invaluable for devised therapeutic interventions.

## DATA AVAILABILITY

BAM files containing trimmed and filtered data for the unsynchronised *LIG3^−/−^:LIG4^−/−^* HCT116 sample are available in the ArrayExpress database (www.ebi.ac.uk/arrayexpress) under accession number E-MTAB-3811.

BAM files containing trimmed and filtered data for the G2-arrested *LIG3^−/−^:LIG4^−/−^* HCT116 sample are available as a NCBI Bioproject (www.ncbi.nlm.nih.gov/bioproject), accession number PRJNA480939.

## Supplementary Material

Supplementary Data
