# Supplementary material for: DNA Ligase 1 is an essential mediator of sister chromatid telomere fusions in G2 cell cycle phase
Source: Nucleic Acids Res. 2018 Dec 21;47(5):2402–24. doi: 10.1093/nar/gky1279 (PMC6411840; doi:10.1093/nar/gky1279)
Supplement: Supplementary Data [file gky1279_supplemental_files.pdf]

DNA Ligase 1 is an essential mediator of sister chromatid telomere fusions in G2 cell cycle phase

Kate Liddiard,[1] Brian Ruis,[2] Yinan Kan,[2§] Kez Cleal,[1] Kevin E. Ashelford,[1] Eric A. Hendrickson[2†] and Duncan M. Baird.[1†]

[1]Division of Cancer and Genetics, School of Medicine, Cardiff University, Heath Park, Cardiff, CF14 4XN, UK.

[2]Department of Biochemistry, Molecular Biology, and Biophysics, University of Minnesota, Minneapolis, MN 55455, USA.

[§]Current address: eGenesis Inc., 300 Technology Square, Cambridge, Massachusetts, MA 02139, USA.

[†]These authors contributed equally to this work

*Correspondence email: bairddm@cardiff.ac.uk

Supplementary Figure 1

Generation and validation of an HCT116 conditional *LIG1* deletion cell line. (a) Upper panel shows the targeting strategy employed to generate HCT116 $LIG1^{-/flox:CreERt2}$ cell line using Cre recombinase-mediated recombination (loxP recognition sites marked as filled arrowheads) to excise exon 23 of human *LIG1*. Lower panel depicts the 'floxed' and deleted *LIG1* alleles within a 1 kb sequence window and presents the comparative sequence of relevant domains: exon 22 is represented by a red block, exon 23 in blue and sequences replaced by loxP Cre recognition sites highlighted in yellow. Diagnostic primer binding sites for validation of allelic deletion are indicated as purple arrows. GRCh38 Chr19 genomic positions delineating the start and end of the relevant sequence window are indicated below; the sequence is colour-coded to match the diagrammatic representations above. (b)(i) Representative gel image (3 experiments) comparison of *LIG1* and *YWHAZ* housekeeping gene expression by RT-PCR in unsynchronised (U) and G1 or G2 cell cycle-arrested $LIG1^{-/flox:CreERt2}$ cells treated with 4-OHT (+) or carrier control (-). Control samples were prepared without reverse transcriptase (RT-). A genomic DNA control sample is labelled as G+ and the water blank lane as H-. Molecular weight ladders (kb and 100 bp) were used to identify LIG1 cDNA amplified using Ex22_F and Ex23_R primers at 221 bp and YWHAZ cDNA at 211 bp. Normalised gene expression values (tester/YWHAZ) are shown below the lanes. Confirmation of the cell cycle status of the same cells is presented in (ii). (iii) RT-PCR detection of transcribed *LIG1* exons 5′ (exons 4-5 and exons 17-19) or 3′ (exons 22-23) of the exon targeted for Cre-mediated recombination (exon 23). Cell cycle-arrested and unsynchronised samples cultured in the presence or absence of 4-OHT were processed as in (i). Data are presented as mean fold change in mRNA expression for 4-OHT/MeOH carrier control-treated samples in two experiments normalised to *YWHAZ* housekeeping gene expression with standard deviation (SD). The red dashes mark 1.0-fold (no) change in gene expression. (c) Representative western blot analysis (2 experiments) demonstrating substantial reduction in LIG1 protein (anti-LIG1 C-terminus polyclonal, upper panel; anti-LIG1 10H5 monoclonal, bottom panel) expression in 4-OHT-treated $LIG1^{-/flox:CreERt2}$ ($L1^{-/fx:Cre}$) and $LIG3^{-/-}$:$LIG4^{-/-}LIG1^{-/flox:CreERt2}$ ($L3^{-/-}$:$L4^{-/-}L1^{-fx:Cre}$) clones C15-5 and C15-9 (see Supplementary Figure 4) but not WT, $LIG3^{-/-}$:$LIG4^{-/-}$ ($L3^{-/-}$:$L4^{-/-}$) or parental C15 $LIG3^{-/-}$:$LIG4^{-/-}LIG1^{-/flox}$ ($L3^{-/-}$:$L4^{-/-}L1^{-fx}$) cells. LIG1 protein signal quantification ($x10^6$) is indicated above the appropriate bands in red. The central panel shows actin protein loading signal control. The lower bar chart displays the mean fold change (with standard error) in aggregated LIG1 protein expression detected with the C-terminal polyclonal and 10H5 monoclonal antibodies normalised to actin and constitutive protein loading signal determined by immunoblotting and membrane staining in 4-OHT-treated compared with MeOH-treated samples. The red dashes mark 1.0-fold (no) change in protein expression. Statistical significance was assessed by one-tailed paired T-tests. (d)(i) Cell cycle analysis of single nuclei suspensions prepared from MeOH or 4-OHT-treated WT and $L1^{-/fx:Cre}$ cells performed by flow cytometry detection of phospho-histone H3 (pHH3)-expressing fractions (blue overlay, positive fractions marked with red bar and indicated as percentages in red). (ii) DRAQ5™ DNA staining of the same samples presented in (i). Diploid nuclei with 2N DNA content and tetraploid nuclei

with 4N DNA content and the relative population proportions are annotated. (e) The specific impact of 4-OHT on the proliferation of *L1*$^{-/fx:Cre}$ and not *L3*$^{-/-}$:*L4*$^{-/-}$*L1*$^{-fx}$, WT or *L3*$^{-/-}$:*L4*$^{-/-}$ cells is demonstrated as a chart of fold change in cell number over 24 hr following treatment with 4-OHT or MeOH carrier. (f) Confirmation of G2-arrest induced by 50ng/ml nocodazole in *L1*$^{-/fx:Cre}$ cells over 24 hr. Upper panels show representative linear DAPI DNA stain signal intensity histograms with diagnostic DNA content peaks labelled. The lower panel shows mean population fraction data from 2 replica experiments plotted as a stacked bar chart with SD. Fold changes in cell cycle fractions with 4-OHT or nocodazole treatment are listed in the table below, with statistical significance between sample populations tested using unpaired one-tailed T-tests. (g) Prototypical images of unsynchronised or G2-arrested (h) WT, *L3*$^{-/-}$:*L4*$^{-/-}$ and *L1*$^{-/fx:Cre}$ cells treated with 4-OHT for 24 hr is shown. Complete field of view images with 20X magnification are displayed with a 250 μM scale bar. (i) Two independent cultures of *L1*$^{-/fx:Cre}$ cells were treated with MeOH or 4-OHT for 24 hr ahead of 7 days' long term culture in standard medium. Prototypical images with 20X magnification and a 250 μM scale bar are shown in (i), fold change in population parameters over 7 days (4-OHT-/MeOH-treated) is shown in (ii), genomic PCR confirmation of *LIG1* allelic recombination is presented in (iii) with quantification of the deleted/'floxed' signal intensity (x10^{-2}) indicated above the appropriate bands in red. RT-PCR analysis of ligase (*LIG1* Ex22_F and Ex23_R primers) and senescence-associated genes normalised to *YWHAZ* housekeeping gene control is displayed as fold change of 4-OHT-/MeOH-treated cells in (iv).

Supplementary Figure 2

LIG1 plays a non-redundant role in sister chromatid telomere fusion. (a) Fusions induced by 17p and 21q TALEN (TLN) pairs in unsynchronised and G2-arrested $L3^{-/-}$:$L4^{-/-}$ cells (left panels) juxtaposed with data for the 4-OHT-treated $L1^{-/fx:Cre}$ (right panels) formerly presented in Figure 2 (a). 17p telomere fusions (upper panel) and 21q telomere fusions (lower panel) were visualised with specific hybridisation probes. (b) 17p telomere sister chromatid telomere fusions amplified by single 17p primer fusion PCR from unsynchronised (upper) and G2-arrested (lower) 17p and 21q TLN-transfected carrier and 4-OHT-treated $LIG1^{-/flox:CreERt2}$ ($L1^{-/fx:Cre}$) cells prepared as in (a) and detected using the 17p subtelomere probe. (c) Transfection efficiency of WT, $L3^{-/-}$:$L4^{-/-}$ and $L1^{-/fx:Cre}$ G2-arrested cells measured as proportions of viable GFP-expressing cells at 24 hr post-nucleofection. Mean proportions calculated from 2-5 independent experiments are plotted with SD and statistical significance assessed by unpaired one-tailed T-tests with Welch's correction applied for the combined WT versus $L1^{-/fx:Cre}$ tests. (d) Titration of DNA input of LIG1-depleted sample into 17p single primer telomere fusion PCR. Input gradient illustrated with black triangle and ng DNA amounts listed above lanes. Abundant 17p fusions amplified using 100 ng/reaction HCT116 WT DNA are shown for comparison (centre). An independent replica of paired carrier and 4-OHT-treated $L1^{-/fx:Cre}$ carrier using 100 ng DNA/fusion PCR was performed in parallel (right-hand lanes). (e) Southern hybridisation images of 17p (upper panel) and 21q (lower panel) TLN-induced fusion amplicons derived from two replica (adjacent lanes) samples of unsynchronised WT HCT116 generated as in (a). (f)(i) Confirmation of L82 LIG1 inhibitor functional activity in HCT116 WT, $L3^{-/-}$:$L4^{-/-}$ and $TP53^{-/-}$ cells. Cells were cultured under conditions of serum starvation (0.1% FCS) for 6 days before being recovered into 20% FCS-containing medium supplemented with 5 μM L82 or carrier control (untreated) for a further 12 or 24 hr, as indicated (t12, t24). WT flow cytometry detection of cells were used to calibrate the phospho-histone H3 (pHH3)-expressing G2/M phase mitotic fractions (percentages indicated in red) in experimental samples (central dot plots) compared with no antibody (no ab) and untreated controls. The lower panels show the overlaid proportions of pHH3-expressing mitotic cells in 24 hr untreated (black) or L82-treated (red) samples. The L82-induced fold changes (L82/untreated) in pHH3-expressing mitotic fractions in t12 (left) and t24 (right) WT (white), $L3^{-/-}L4^{-/-}$ (grey) and $TP53^{-/-}$ (black) samples are expressed as bar charts below with fold-change values in red. (ii) Validation of L82-mediated inhibition of LIG1-dependent cell cycle progression in $L3^{-/-}$:$L4^{-/-}$ cells. $L3^{-/-}$:$L4^{-/-}$ cells were counted and plated 24 hr prior to being treated with 50 μM L82 or DMSO carrier control for an additional 24 or 48 hr in two experiments. Cells harvested at 24 and 48 hr were counted and processed for cell cycle analysis. The upper chart shows population growth and the central panel shows altered cell cycle distributions in response to the treatments. The lower panel specifically charts the mean change in S-phase fractions after 24 and 48 hr plotted with SD.

(g) (i) Southern hybridisations of 17p (upper panel) and 21q (lower panel) telomere fusions amplified from 50 μM L82-treated *L3*$^{-/-}$*:L4*$^{-/-}$ cells 48 hr post-nucleofection with 17p and 21q TLN or 0 DNA controls are shown. (ii) 17p (left) and 21q (right) fusion frequencies calculated from 3 independent experiments are summarised as means with 95% confidence intervals and differences tested using one-tailed paired T-tests.

Supplementary Figure 3

LIG1-depleted cells display an intact DNA damage response. (a) The expression of senescence-associated (*DPP4* and *HMGA2*; 3 replicas) and TP53-activated (*CDKN1A*; 2 replicas) genes in unsynchronised and G2- or G1-arrested $L1^{-/fx:Cre}$ cells harvested after 24 hr was analysed by RT-PCR and displayed as mean fold change with SD of 4-OHT-/MeOH-treated cells normalised to *YWHAZ* housekeeping gene expression. The red dotted line is set at 1.0-fold (no change). (b) Ploidy distributions are not significantly altered by 17p TLN transfection in G2-arrested LIG1-depleted cells. The bar chart shows separated data for 17p TLN or 0 DNA control transfected $L1^{-/fx:Cre}$ cells harvested at 24 hr post-nucleofection. The proportions of cells in each cell cycle phase are colour coded as in Figure 3b and 3c. Data for each fraction is plotted as the mean with SD. One-way non- Kruskal-Wallis ANOVA of individual population fractions (significance set as alpha = 0.05) indicates no statistically significant differences; *P* values listed alongside relevant populations.

Supplementary Figure 4

Construction and validation of an HCT116 *LIG3*$^{-/-}$:*LIG4*$^{-/-}$ conditional *LIG1* deletion cell line. (a) Exon 23 (blue) of one *LIG1* allele was targeted by CRISPR/Cas9. Flanking Cre recombinase loxP sites (yellow arrowheads) were introduced by homology-directed repair following electroporation of a rAAV plasmid donor bearing a silent mutation that interrupts the protospacer adjacent motif (PAM) sequence to prevent donor plasmid cleavage by the Cas9 nuclease. One clone (#15; C15) from 21 screened was identified with one correctly 'floxed' allele and one null allele with an exon 23 partial 3' deletion that includes the splice donor for exon 24 (*LIG3*$^{-/-}$:*LIG4*$^{-/-}$*LIG1*$^{-/flox}$). Lentiviral integration of Cre-oestrogen receptor (CreERt2) transgene generated a stable conditional *LIG1* deletion cell line (*LIG3*$^{-/-}$:*LIG4*$^{-/-}$*LIG1*$^{-/flox:CreERt2}$) where excision of the single remaining functional exon 23 is achieved by treatment with 4-OHT. Two clones (C15-5 and C15-9) were isolated and validated for use in these experiments. The lower panel shows a 640 bp sequence window and cartoon depiction (above) of the human *LIG1* targeted locus displaying the *LIG1* sequence replaced by loxP sites (yellow) flanking exon 23 (blue) with CRISPR/Cas9 sgRNA recognition site marked with a grey box. Diagnostic primer binding sites for validation of allelic deletion are indicated as purple arrows and within the text sequence as purple underlined (LIG1 Ex22_F) or highlighted (LIG1 Int23_R) sequences. GRCh38 Chr19 genomic positions delineating the start and end of sequence window are indicated in bold. The *LIG1* null allele created by the initial CRISPR/Cas9 targeting is displayed in (b) aligned with the endogenous *LIG1* sequence (WT). Exon 23 sequence in blue capitals, intron 23-24 sequence in dark grey lower case, deleted nucleotides represented by dashes. The bottom line of sequence shows the continuous null allele sequence resulting from the deletions. (c) (i) Diagram of *LIG1* allelic recombination outcomes: genomic (WT); 'floxed' allele with intact exon 23 (blue) flanked with loxP sites (yellow arrowheads); null allele with exon 23 partial deletion (abridged blue exon 23 box); deleted allele following 4-OHT-activated Cre recombination of exon 23 flanking loxP sites. (ii) Genomic PCR results obtained using primers indicated in (a) to amplify *LIG1* locus from *LIG3*$^{-/-}$:*LIG4*$^{-/-}$:*LIG1*$^{-/flox}$ (C15) and *LIG3*$^{-/-}$:*LIG4*$^{-/-}$:*LIG1*$^{-/flox:CreERt2}$ (C15-5 and C15-9) cells treated with MeOH carrier (-) or 4-OHT (+) for 24 hr. Band signal intensity values for null (N) and deleted/'floxed' (Δ/Fx) allelic ratios (x10^{-2}) are listed above the sample lanes. G+; WT HCT116 and H-; water blank controls. (iii) Mean relative expression of LIG1 mRNA (Ex22_F and Ex23_R primers) in MeOH- and 4-OHT-treated *LIG3*$^{-/-}$:*LIG4*$^{-/-}$:*LIG1*$^{-/flox:CreERt2}$ cells, as determined by RT-PCR and plotted with 95% confidence intervals. Statistical significance was assessed by one-tailed paired T-test with *P* value reported. (d)(i) Genomic PCR results obtained as in (c)(ii) in unsynchronised (Noc -) or G2-arrested (Noc +) *LIG3*$^{-/-}$:*LIG4*$^{-/-}$:*LIG1*$^{-/flox}$ (C15) and *LIG3*$^{-/-}$:*LIG4*$^{-/-}$:*LIG1*$^{-/flox:CreERt2}$ (C15-5 and C15-9) cells treated with MeOH carrier (-) or 4-OHT (+) for 24 hr. Band signal intensity values for null (N) and deleted/'floxed' (Δ/Fx) allelic ratios (x10^{-2}) are listed above the sample lanes. G+; WT HCT116 and H-; water blank controls. (ii) Prototypical images of unsynchronised or G2-arrested *LIG3*$^{-/-}$:*LIG4*$^{-/-}$:*LIG1*$^{-/flox}$ (C15) and *LIG3*$^{-/-}$:*LIG4*$^{-/-}$:*LIG1*$^{-/flox:CreERt2}$ (C15-5 and C15-9) cells treated with 4-OHT for 24 hr is shown. Complete field of view images with 20X magnification are displayed with a 250 μM scale bar. (iii) Changes in mean cell diameter induced by 24 hr

MeOH (black shapes) or 4-OHT (white shapes) treatment of G2-arrested *LIG3*$^{-/-}$*:LIG4*$^{-/-}$*:LIG1*$^{-/flox}$ (C15) and *LIG3*$^{-/-}$*:LIG4*$^{-/-}$*:LIG1*$^{-/flox:CreERt2}$ (C15-5 and C15-9) cells from 3-5 independent experiments are shown as mean values with 95% confidence intervals. One-tailed paired T-tests between appropriate samples were performed to test statistical significance.

Supplementary Figure 5

Fusions amplified from LIG1-depleted *LIG3$^{-/-}$:LIG4$^{-/-}$:LIG1$^{-/flox:CreERt2}$* cells are characterised by fragmented insertion and inversions. Examples of 21q TLN-induced telomere fusions reamplified from G2-arrested and 4-OHT-treated *LIG3$^{-/-}$:LIG4$^{-/-}$:LIG1$^{-/flox:CreERt2}$* (Clone 15-9) cells and Sanger sequenced are shown in (i)-(iv). The (shorter) centromeric and longer (telomeric) components of each fusion are depicted in red and blue, respectively, in accordance with Figure 4. The distance of the fusion point from the 21q TLN cleavage site (1.5kb from the start of the telomere repeat array) is marked as ∆bp in the appropriate colours. Microhomology at the fusion junction is indicated in purple and underlined. Insertions are depicted in green and localised templated duplications are in italics and underlined. Fusion sequence (iv) illustrates complex template switching within a genomic context resulting from a fusion with the 16p subtelomere cleaved by the 21q TLN within 1 kb of the telomere repeat array. Fragments of the *BCL9L* gene on chromosome 11 annealed in opposing orientations are marked in orange and brown and // indicates a break in the alignment.

Supplementary Table 1

Primers used in this study

Primer name	Primer sequence 5′ to 3′	Target gene symbol	Assay usage	cDNA amplicon size	gDNA amplicon size	Annealing temperature (Tm) °C
YWHAZ_F	TTCTTGATCCCCAATGCTTC	*YWHAZ*	RT-PCR	211 bp	828 bp	60
YWHAZ_R	AGTTAAGGGCCAGACCCAGT	*YWHAZ*	RT-PCR	211 bp	828 bp	60
LIG1_Ex4_F	GGAAGAGGAGGATGAAGCCC	*LIG1*	RT-PCR	161bp	4392bp	60
LIG1_Ex5_R	GACGCTTCGGAATCCTGAT	*LIG1*	RT-PCR	161bp	4392bp	60
LIG1_Ex17_F	GAATTATCCCCGTGCTGCT	*LIG1*	RT-PCR	254bp	2947bp	65
LIG1_Ex19_R	CGGGTACTTCCCAGTGTTGT	*LIG1*	RT-PCR	254bp	2947bp	65
LIG1_Ex22_F	CTGGTACGTGAGCCCCTTT	*LIG1*	RT-PCR	221 bp	378 bp	60
LIG1_Ex23_R	AGCCAGTTGTGCGATCTCTT	*LIG1*	RT-PCR	221 bp	378 bp	60
LIG3_F	AGGCAGCAGGTACACCAAAG	*LIG3*	RT-PCR	235 bp	3569 bp	60
LIG3_R	TCCCGTAGCAGACAGTCCTT	*LIG3*	RT-PCR	235 bp	3569 bp	60
LIG4_F	CCGAGGCCAGTTAAACGAG	*LIG4*	RT-PCR	287 bp	3528 bp	60
LIG4_R	TCCATAGGCCATTCTCTCTCT	*LIG4*	RT-PCR	287 bp	3528 bp	60
DPP4_F	CAGCTGACAGTCGCAAAACT	*DPP4*	RT-PCR	229 bp	1926 bp	60
DPP4_R	TGCCCATCAGGAGATATTGA	*DPP4*	RT-PCR	229 bp	1926 bp	60
HMGA2_F	ACTTCAGCCCAGGGACAAC	*HMGA2*	RT-PCR	180 bp	13229 bp	60
HMGA2_R	TCCAGTGGCTTCTGCTTTCT	*HMGA2*	RT-PCR	180 bp	13229 bp	60
CDKN1A_F	GCGACTGTGATGCGCTAAT	*CDKN1A*	RT-PCR	400 bp	1603 bp	60
CDKN1A_R	TAGGGCTTCCTCTTGGAGAA	*CDKN1A*	RT-PCR	400 bp	1603 bp	60
IL6_F	TACCCCCAGGAGAAGATTCC	*IL6*	RT-PCR	175 bp	1232 bp	60
IL6_R	TTTTCTGCCAGTGCCTCTTT	*IL6*	RT-PCR	175 bp	1232 bp	60
LIG1_LOXP_SF	ATGTCCTGTTCGCCTCAC	*LIG1*	Genomic PCR	NA	WT:563 bp<br>Floxed:698 bp<br>Deleted:448 bp	60
LIG1_LOXP_SR2	AGGGTACACAGTGGAGTC	*LIG1*	Genomic PCR	NA	WT:563 bp<br>Floxed:698 bp<br>Deleted:448 bp	60
LIG1 Ex22_F	CTGGAGCAGTCAGTGAAAGG	*LIG1*	Genomic PCR	NA	WT:464 bp<br>Floxed:503 bp<br>Deleted:260 bp	60
LIG1 Int23_R	GGCTGTTCTCCATCAGAACTC	*LIG1*	Genomic PCR	NA	WT:464 bp<br>Floxed:503 bp<br>Deleted:260 bp	60
17p6	GGCTGAACTATAGCCTCTGC	17p subtelomere	Telomere fusion PCR	NA	NA	62
XpYpM	ACCAGGTTTTCCAGTGTGTT	XpYp subtelomere	Telomere fusion PCR	NA	NA	62
21q1	CTTGGTGTCGAGAGAGGTAG	21q family subtelomere	Telomere fusion PCR	NA	NA	62

Supplementary Figure 1

a

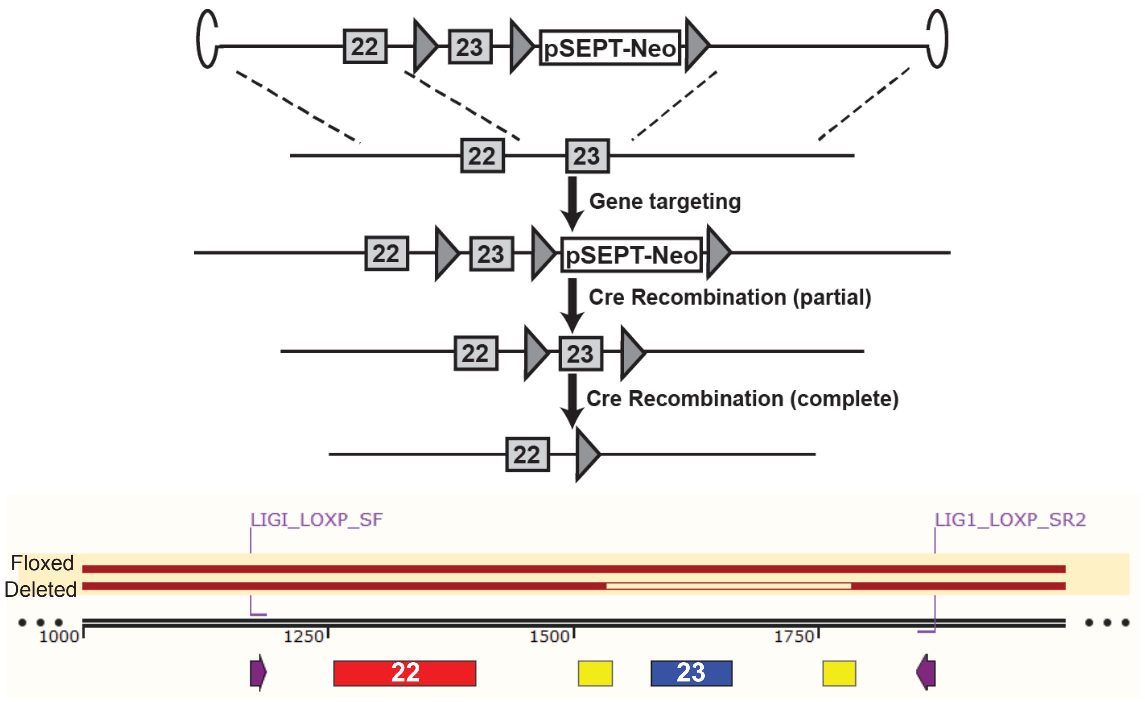

19: 48123673

gactctaaatggtgtcgggccctcttgccagtgactagccccatgctttaagctctcagaatccttctgacatcagggtt
cttccctctcaaggctttccacgaaccctgtcactagttgtgacccgcacatgtcctgttcgcctcacatttagggctta
ttgtctttattctcacaaagatgtctcatctctcttctccctgccggccctgcagtccctggtacgtgagcccctttccc
ggcgccggcagctgctccgggagaactttgtggagacagagggcgagtttgtcttcgccacctccctggacaccaaggac
atcgagcagatcgccgagttcctggagcagtcagtgaaaggtgagggtgctcactttctctcctgaggtctgcagcttg
cgctgaccccggcctcccagcctgtccccagtcctggaccgtttgaccagaatcctgcctgctccataacttcgtatagc
atacattatacgaagttatccagggcttcccaaggaccacgcttctgtgtctccctcagactcctgcgagggctgatgg
tgaagaccctggatgttgatgccacctacgagatcgccaagagatcgcacaactggctcaaggtgattctcgaccccaa
ctgggccttctccaccccgggtctggaggtctgtcccagctaggcagtggatttcgtctagaactagtggatccataact
tcgtatagcatacattatacgaagttatccaaattttaaggtaccactgtgcatatgtatgaattcgatatcaagcttat
cgataccgtcgactccactgtgtaccctgtctgagctcagggcccctttcacaatcccaccccagcccccctgctgcaac
tgagttcagaccttctccctgctgcgagcccttgctcacgagttctgatggagaacagccccagcccaagcaccgcacat

19: 48122709

Supplementary Figure 1

b

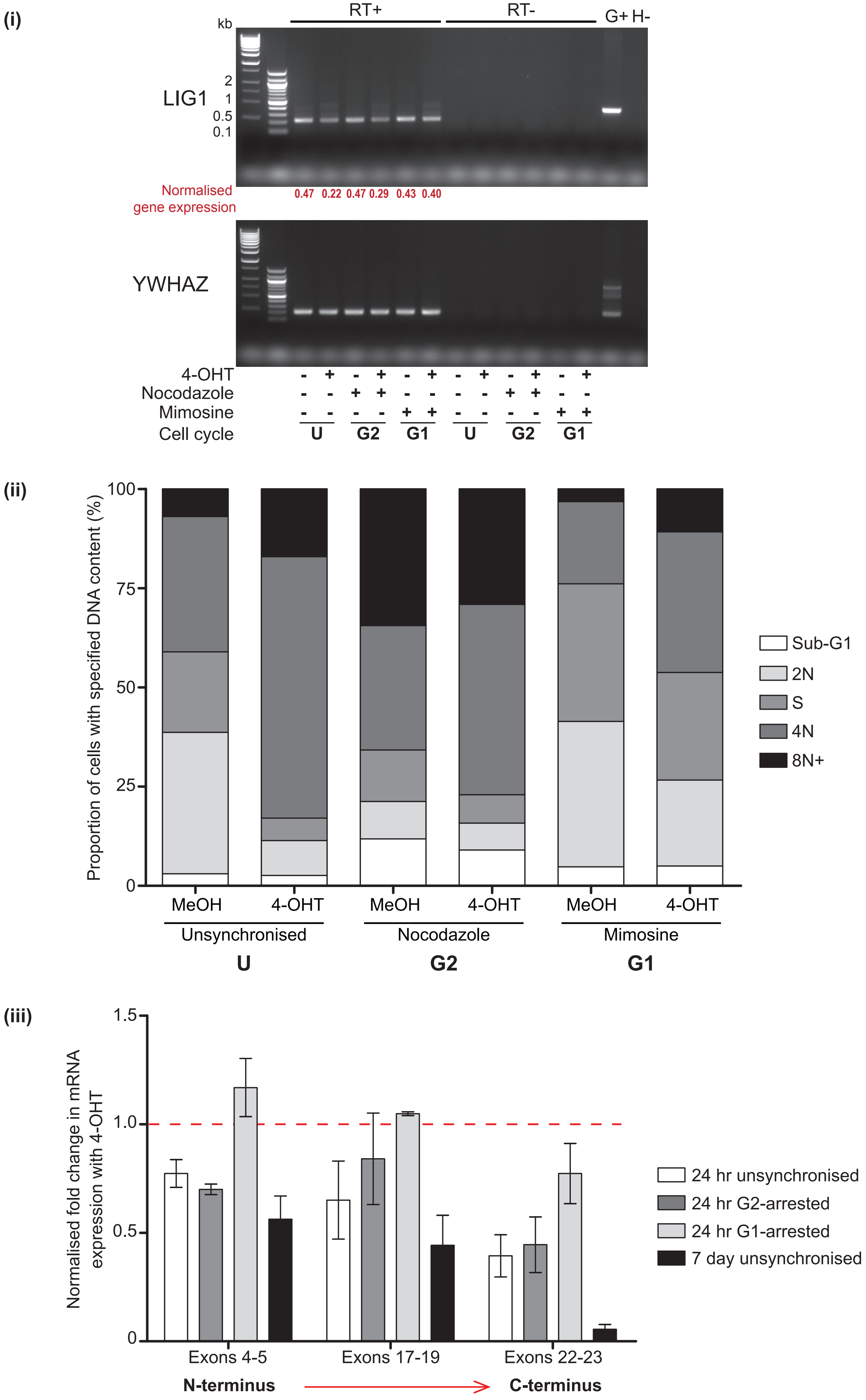

Supplementary Figure 1

c

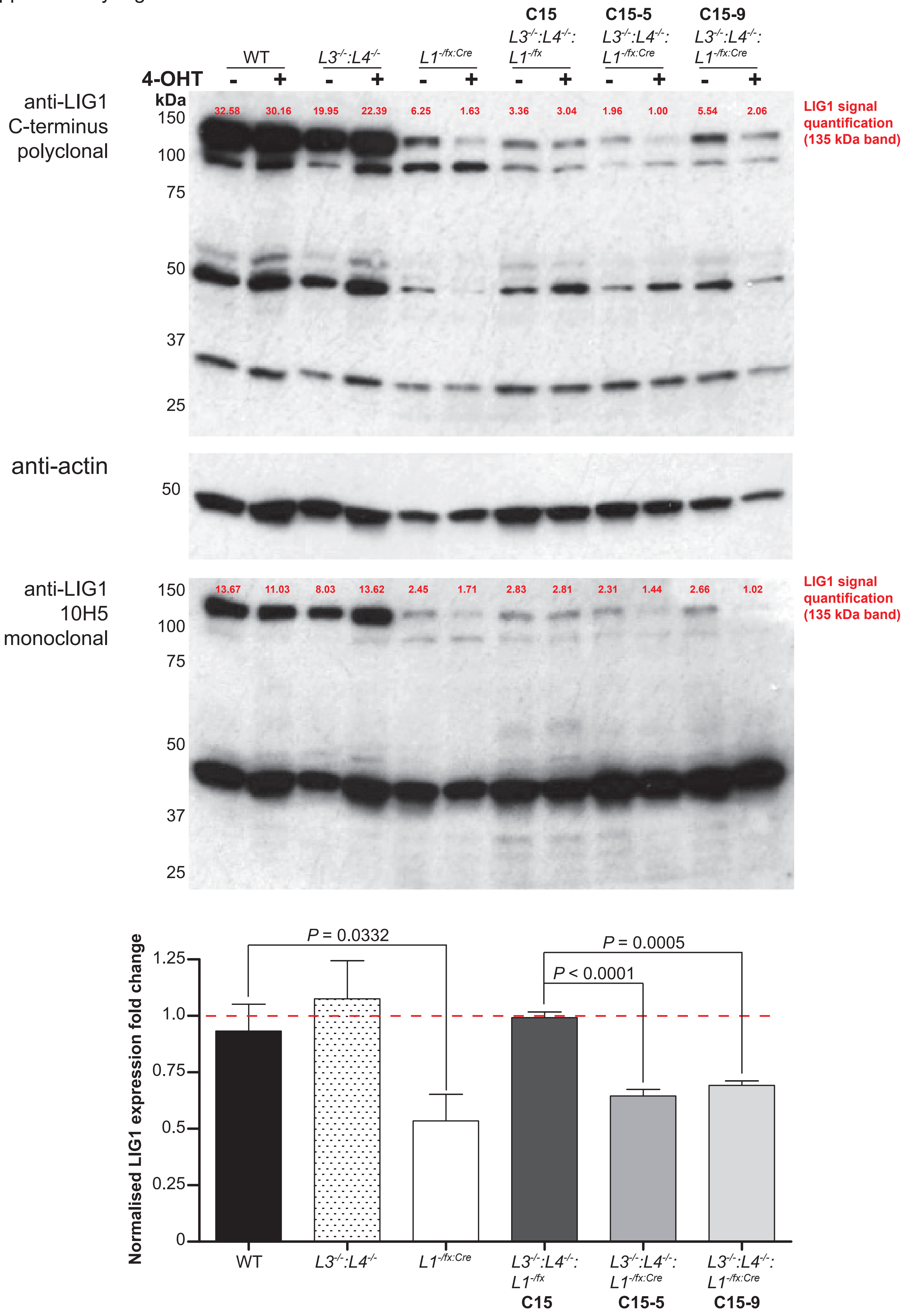

Supplementary Figure 1

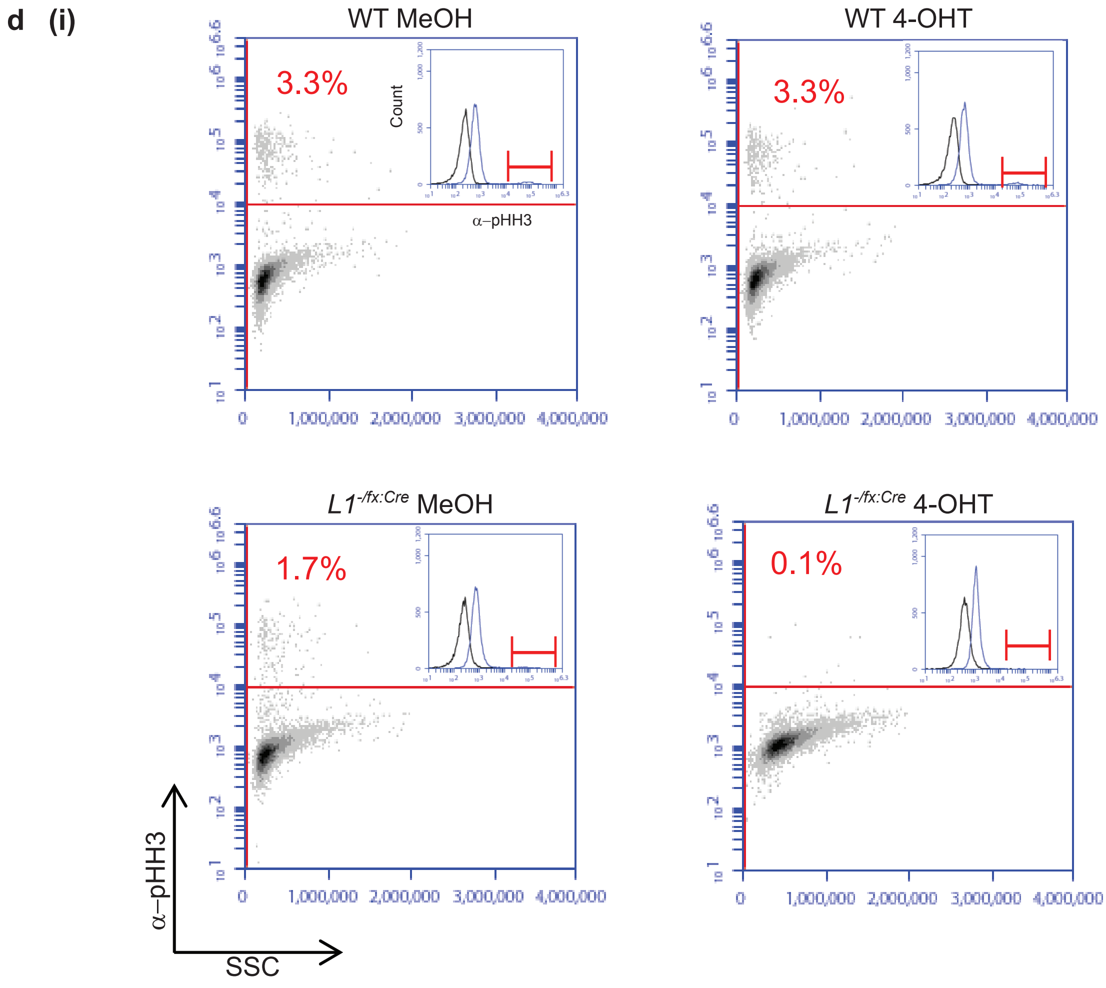

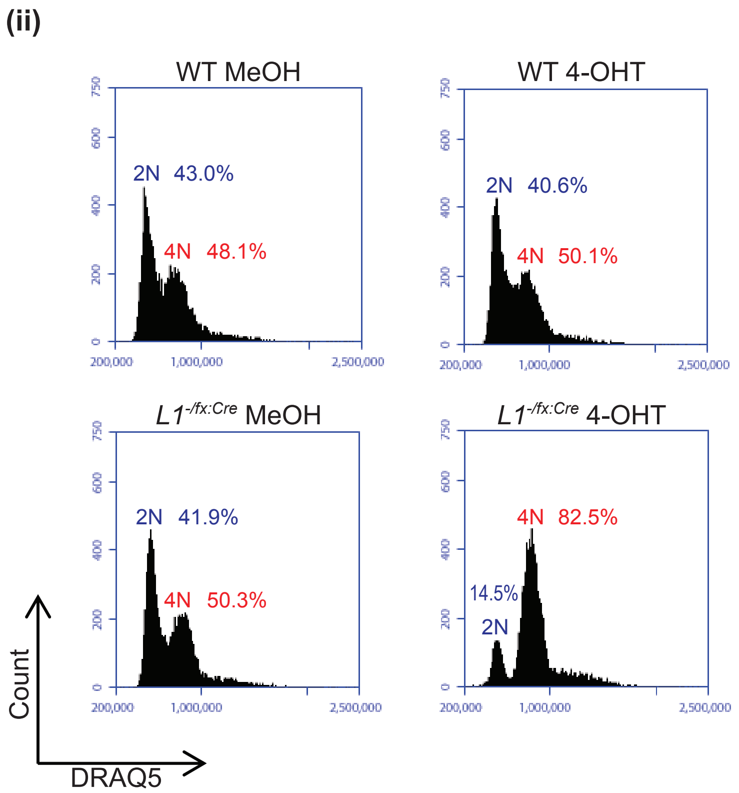

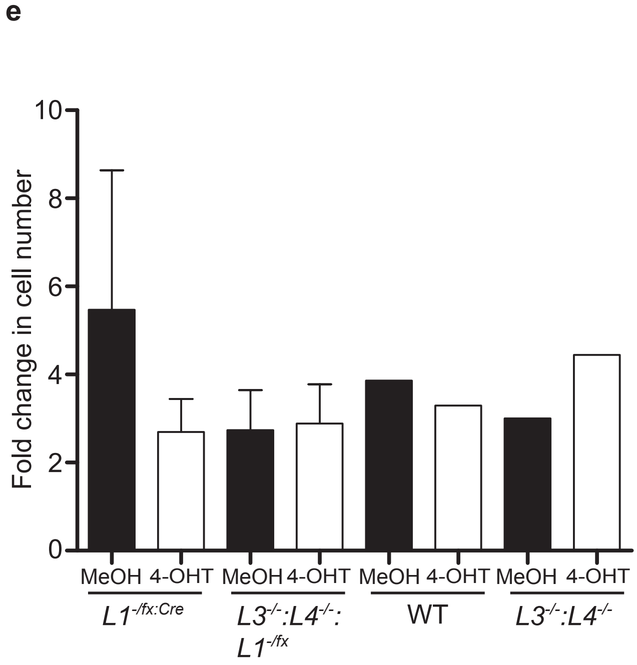

Supplementary Figure 1

f

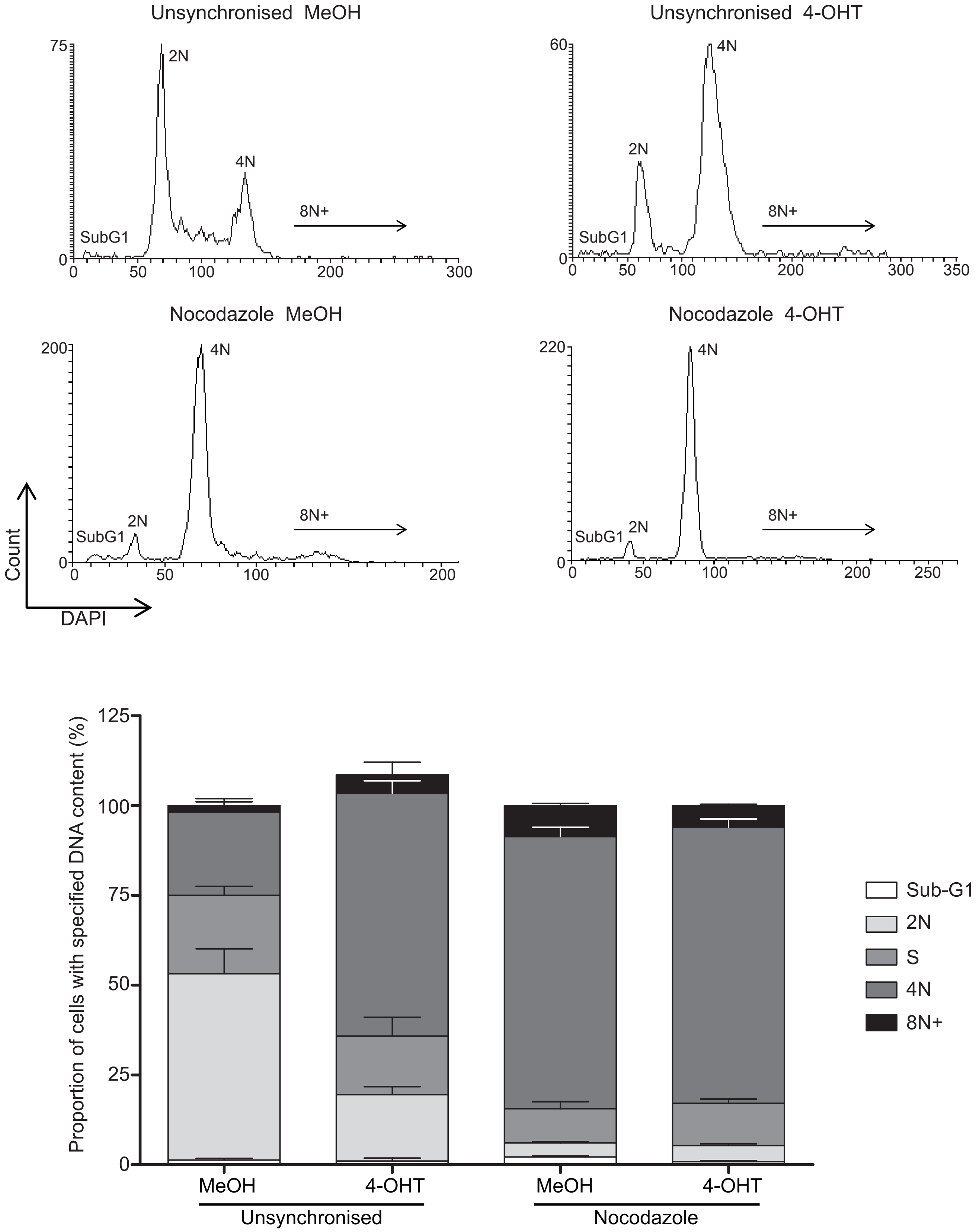

Cell cycle fraction	Comparison	Fold Change	Unpaired T-test *P* value
SubG1	Unsynch. 4-OHT/Unsynch. MeOH	0.79	0.729
SubG1	Nocodazole MeOH/Unsynch. MeOH	1.7	0.731
2N	Unsynch. 4-OHT/Unsynch. MeOH	0.36	0.0114 *
2N	Nocodazole MeOH/Unsynch. MeOH	0.075	0.0051 *
S	Unsynch. 4-OHT/Unsynch. MeOH	0.75	0.157
S	Nocodazole MeOH/Unsynch. MeOH	0.44	0.0161 *
4N	Unsynch. 4-OHT/Unsynch. MeOH	2.92	0.034 *
4N	Nocodazole MeOH/Unsynch. MeOH	3.27	0.0019 *
8N	Unsynch. 4-OHT/Unsynch. MeOH	2.52	0.1661
8N	Nocodazole MeOH/Unsynch. MeOH	4.74	0.0082 *

Supplementary Figure 1

g

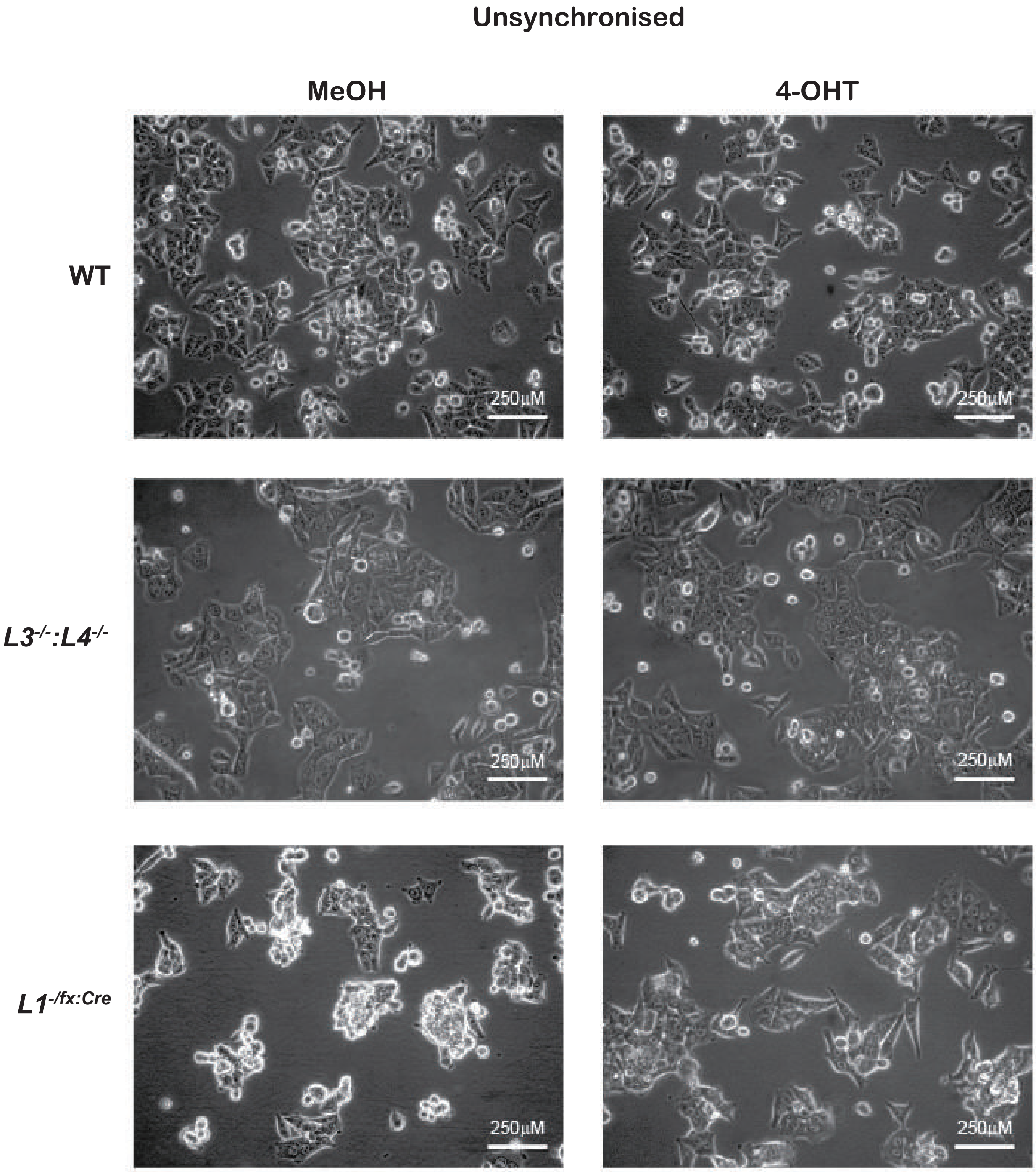

Supplementary Figure 1

h

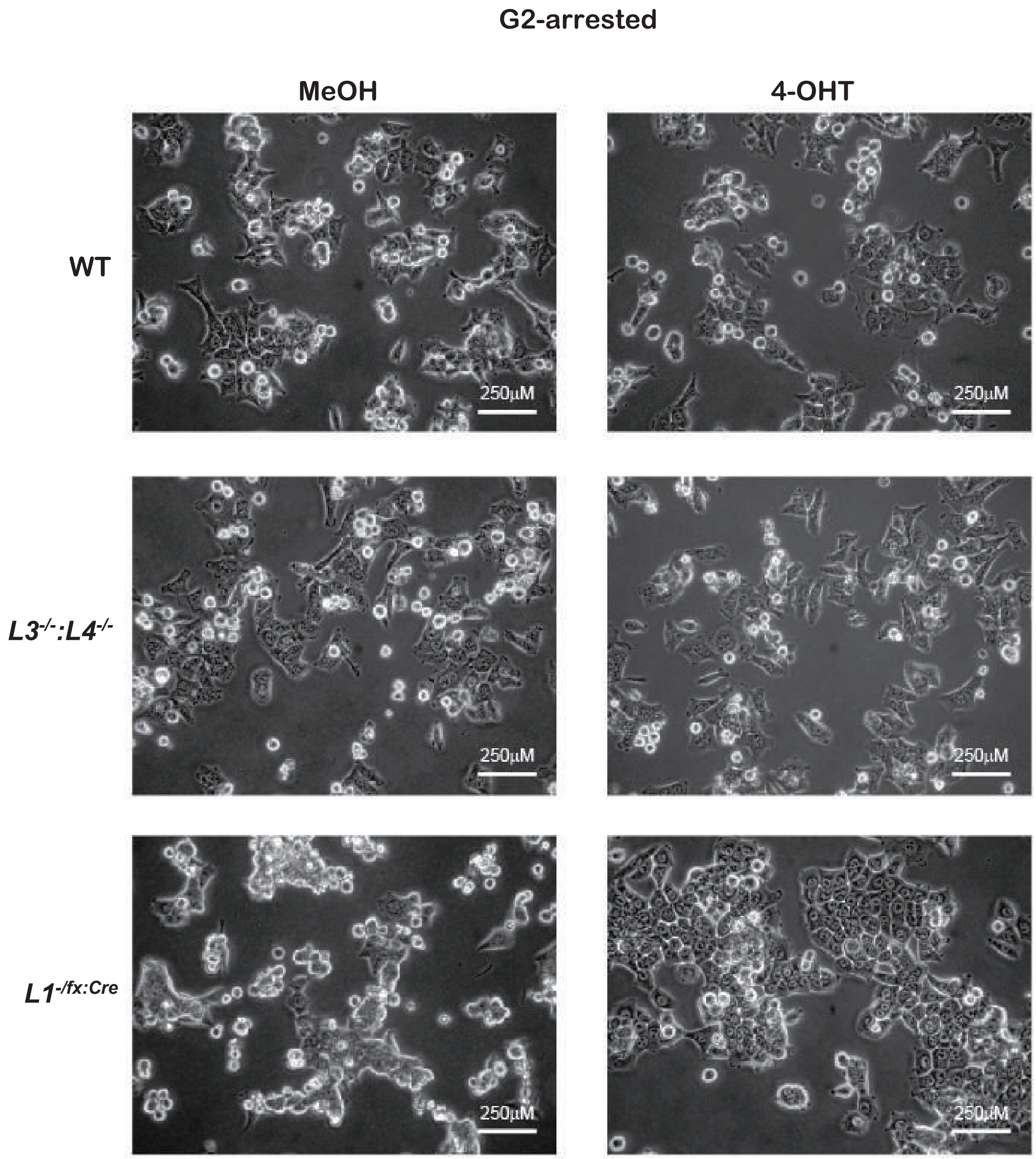

Supplementary Figure 1

i

(i)

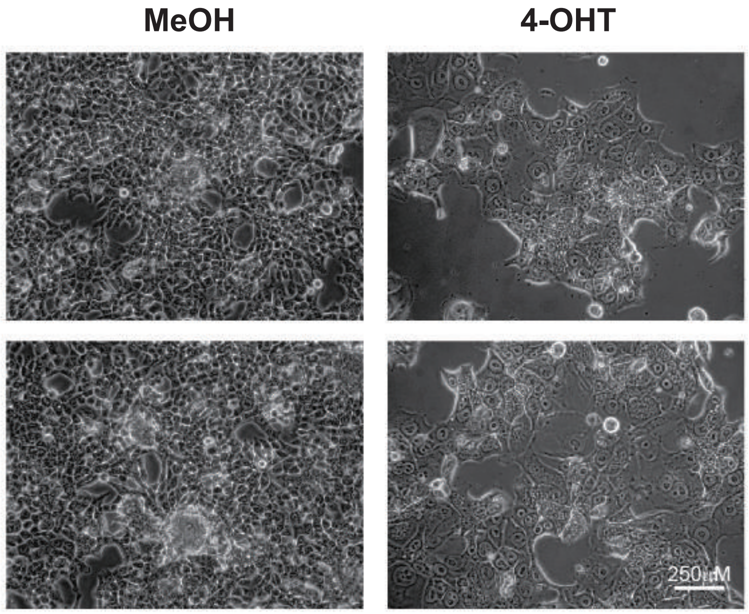

(ii)

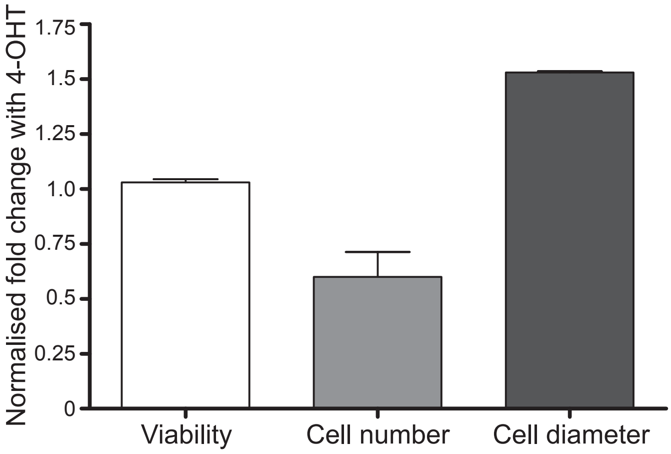

(iii)

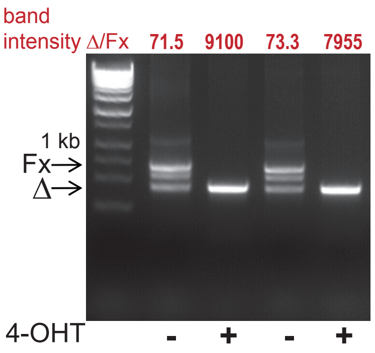

(iv)

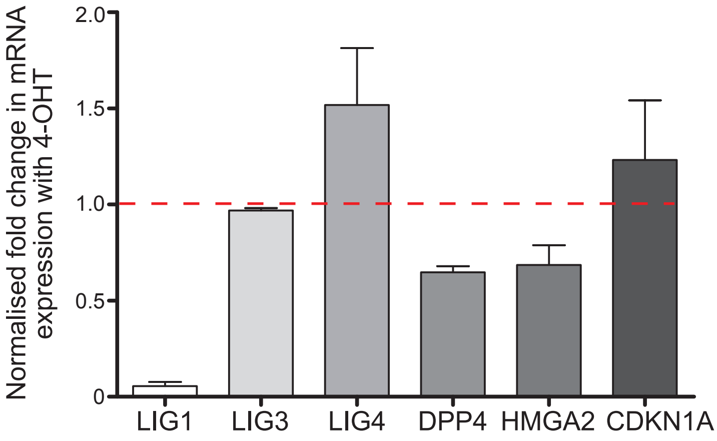

Supplementary Figure 2

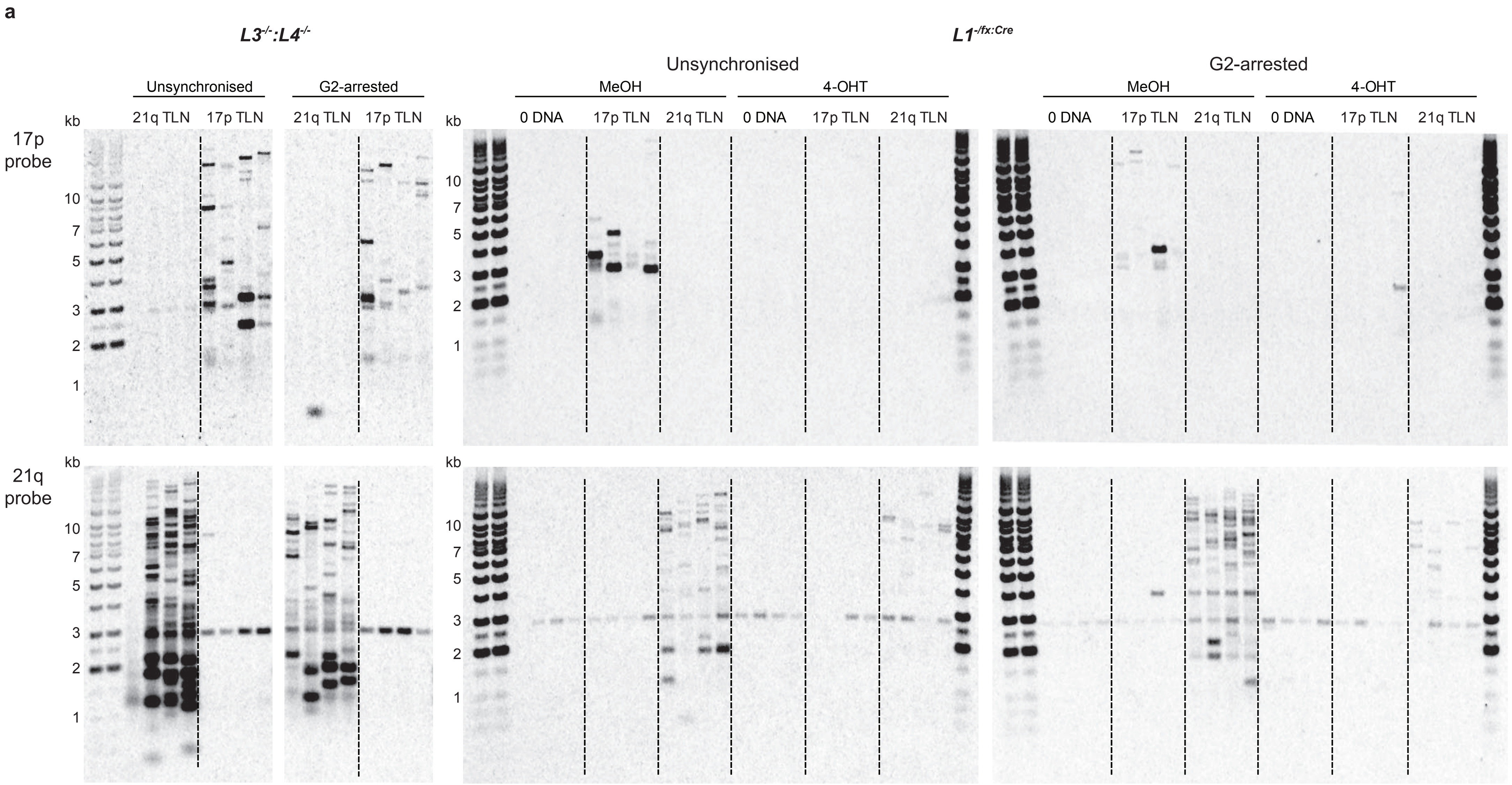

Supplementary Figure 2

b

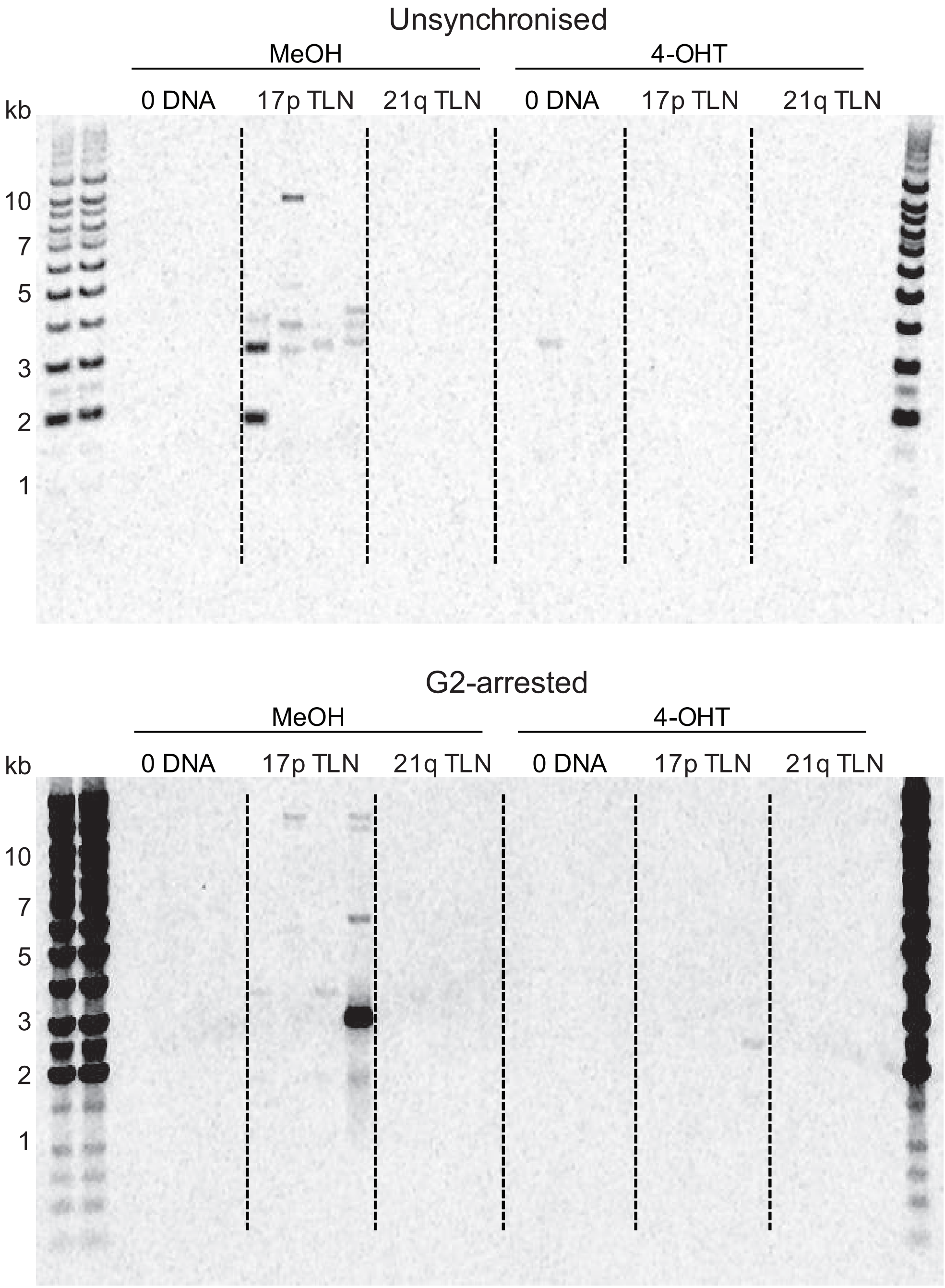

Supplementary Figure 2

c

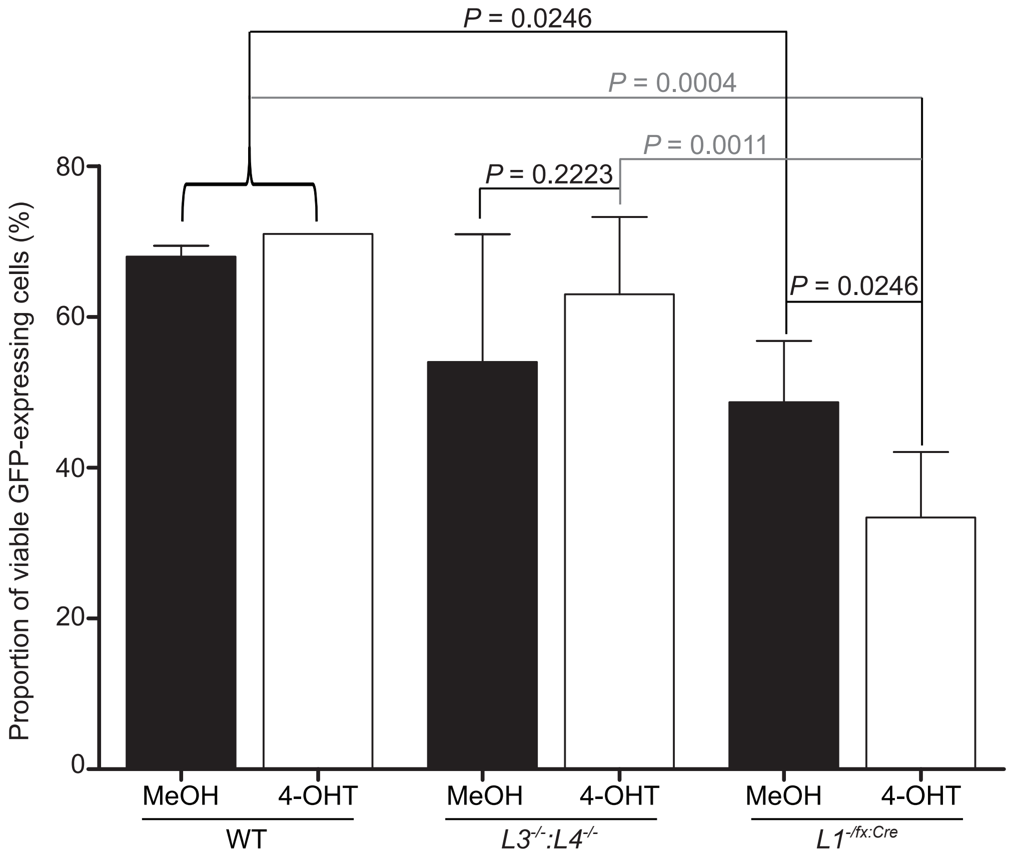

d

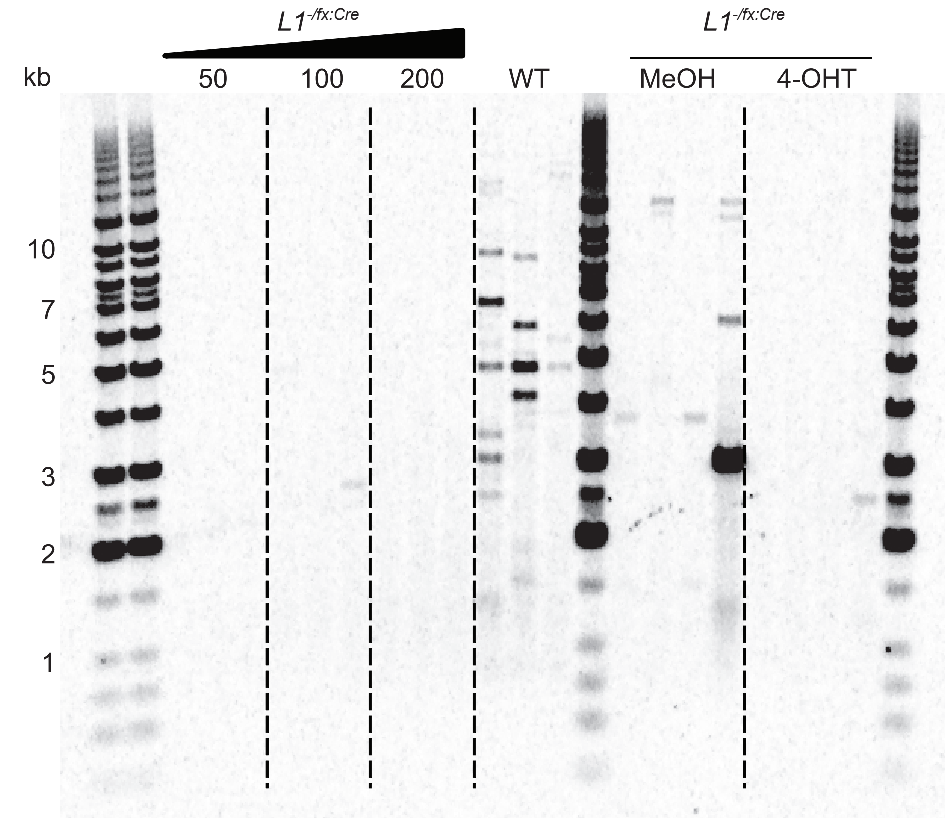

Supplementary Figure 2

e

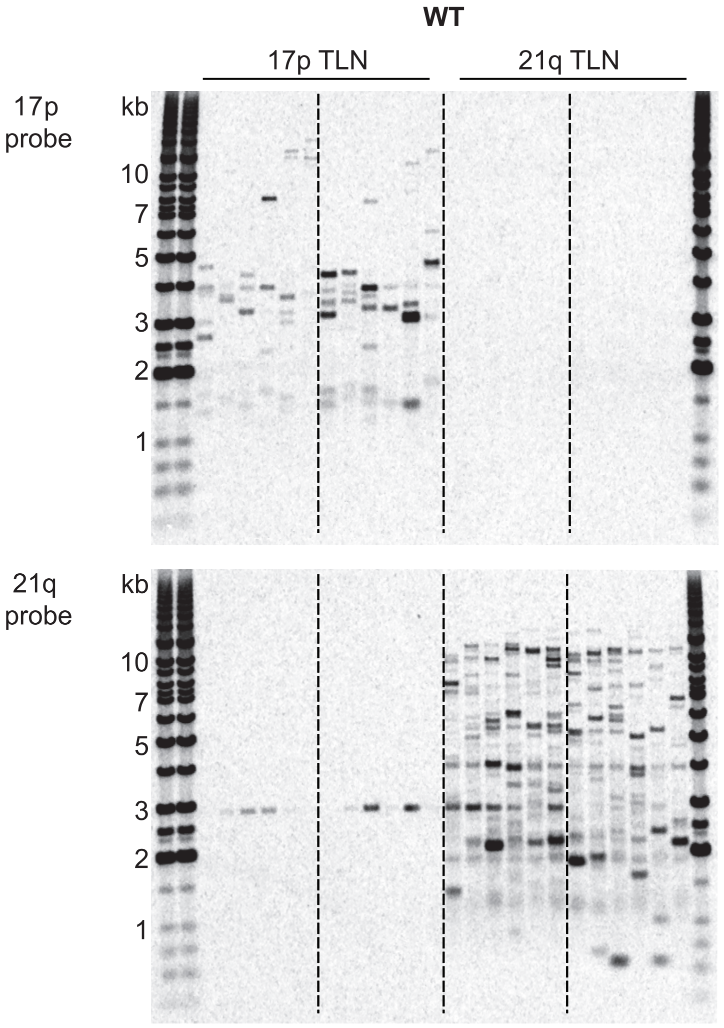

Supplementary Figure 2

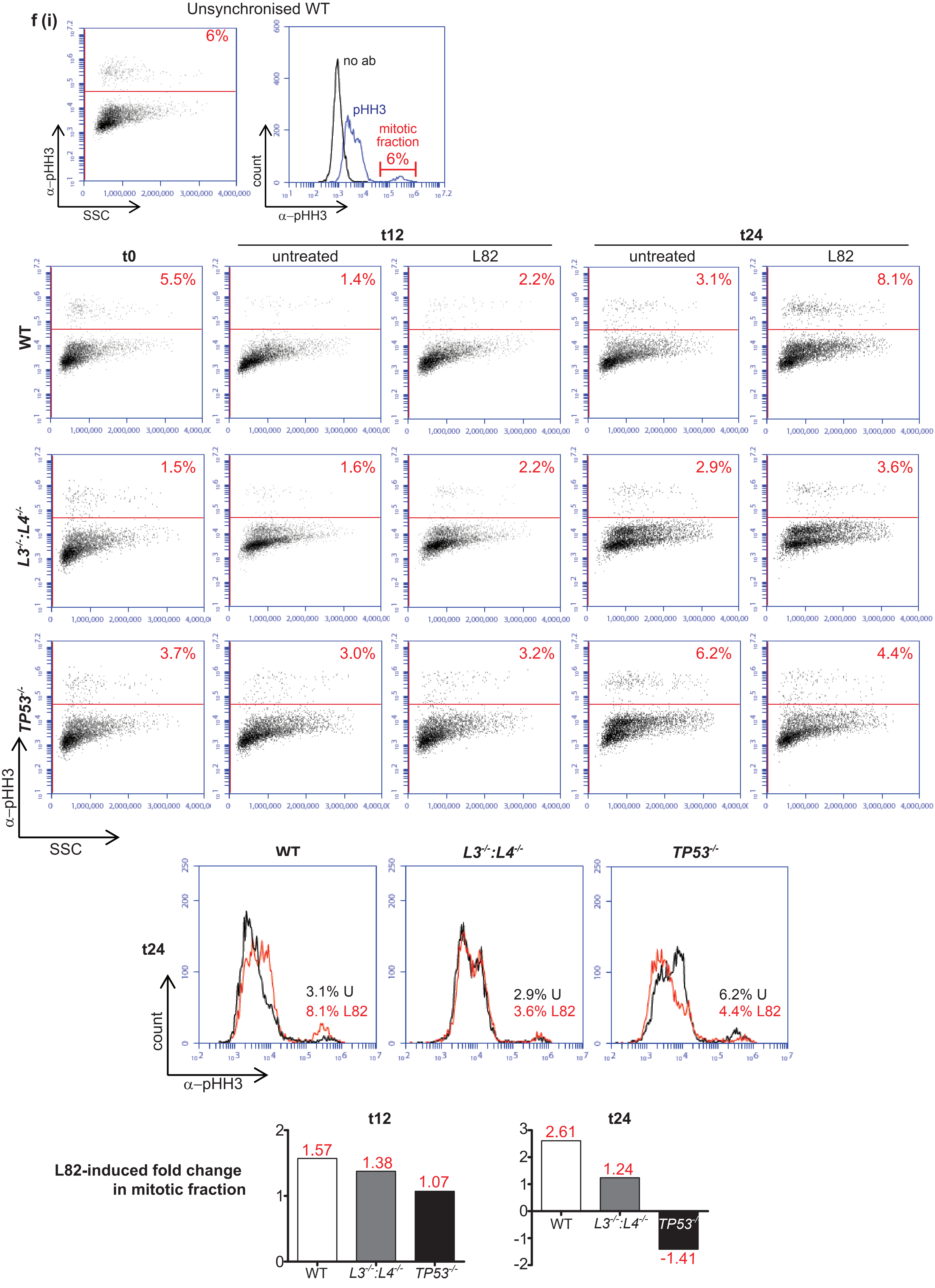

Supplementary Figure 2

f (ii)

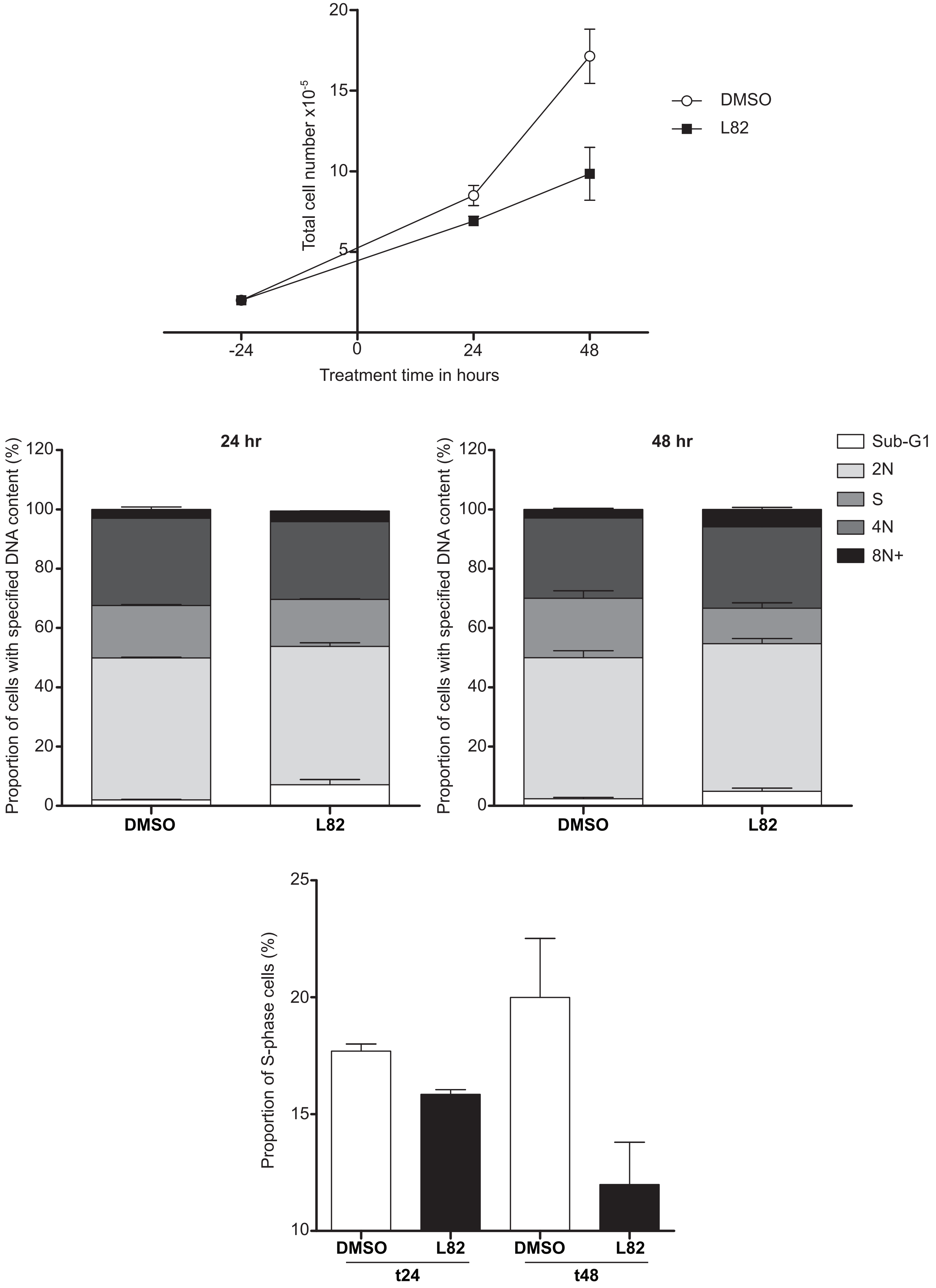

Supplementary Figure 2

g (i)

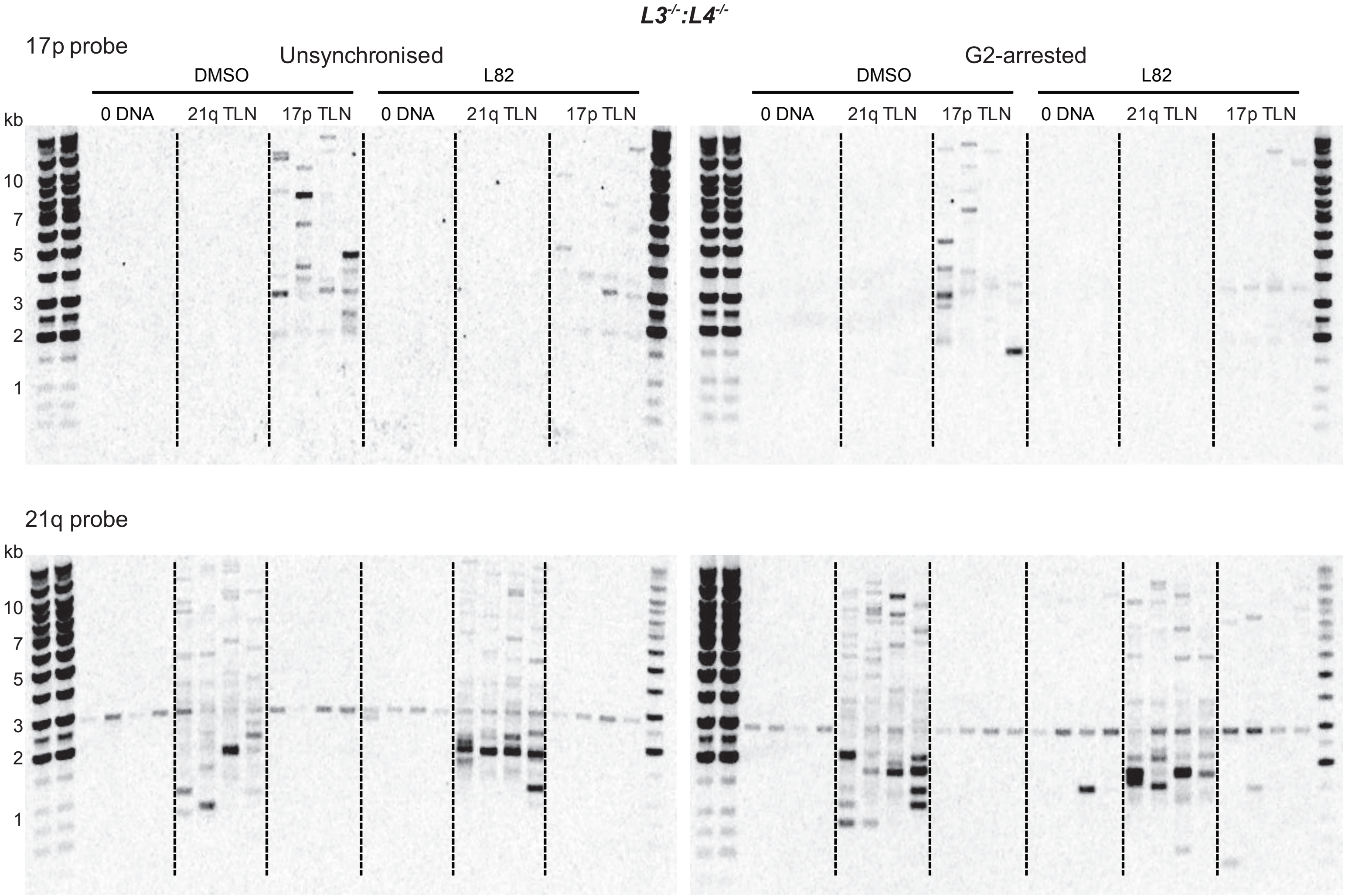

(ii)

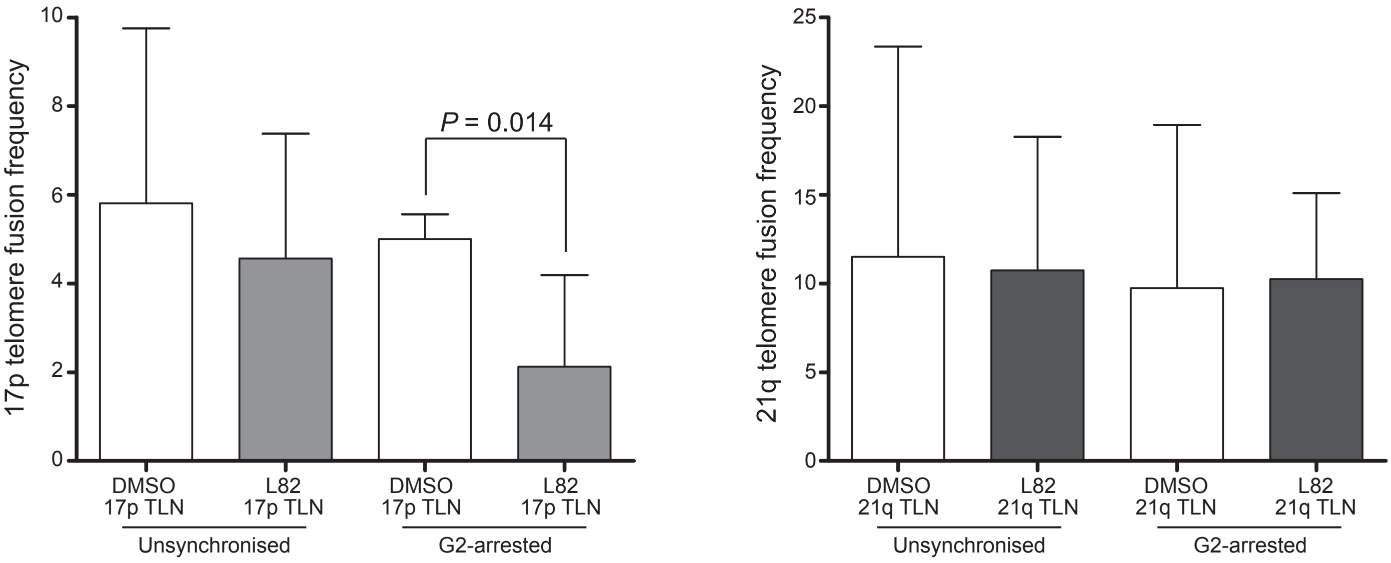

Supplementary Figure 3

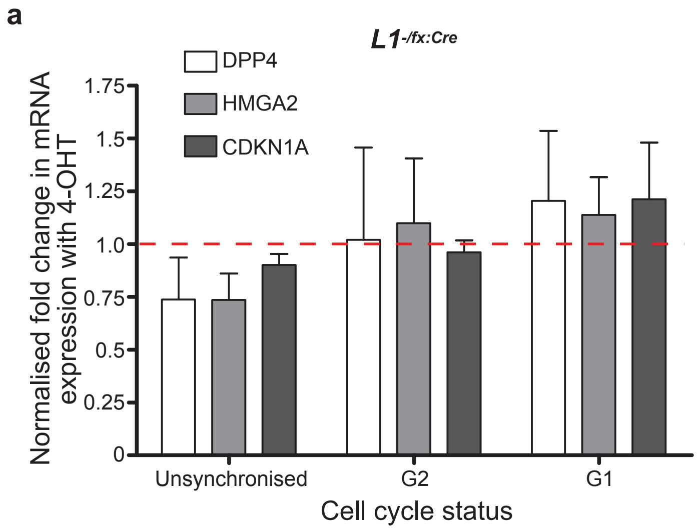

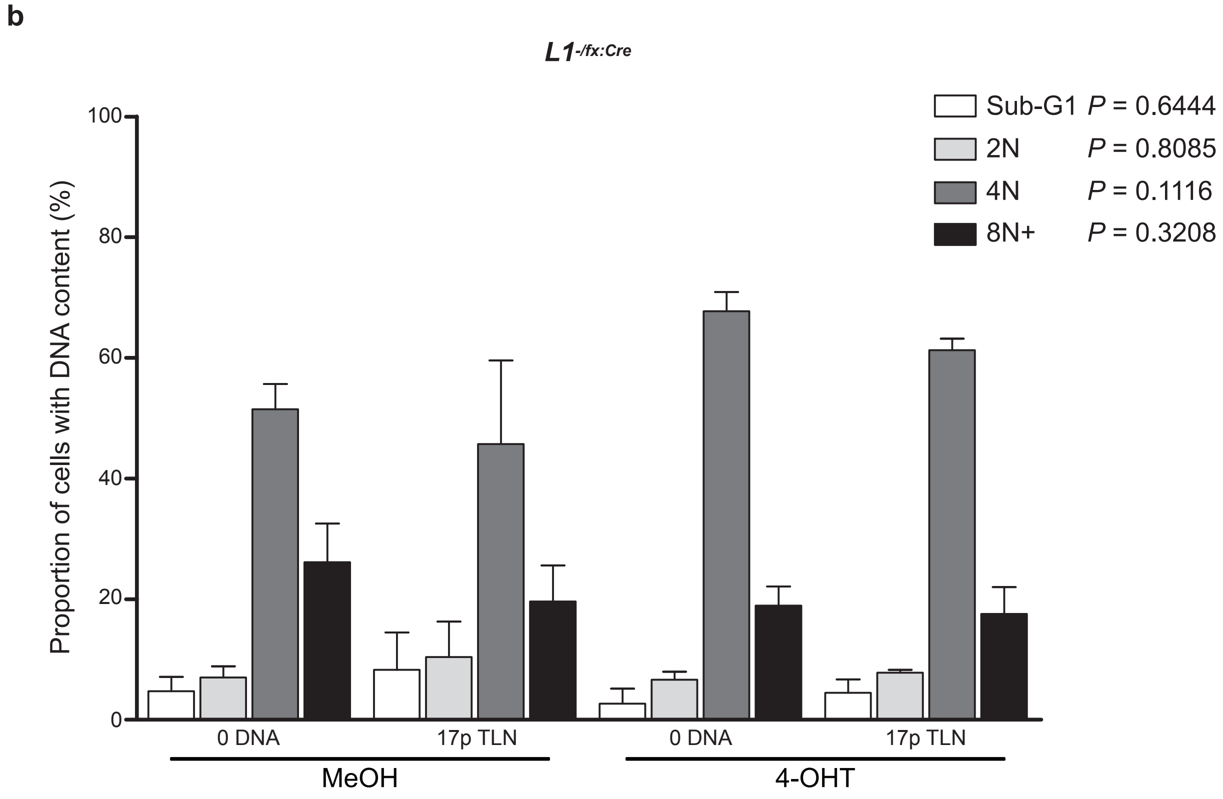

Supplementary Figure 4

a

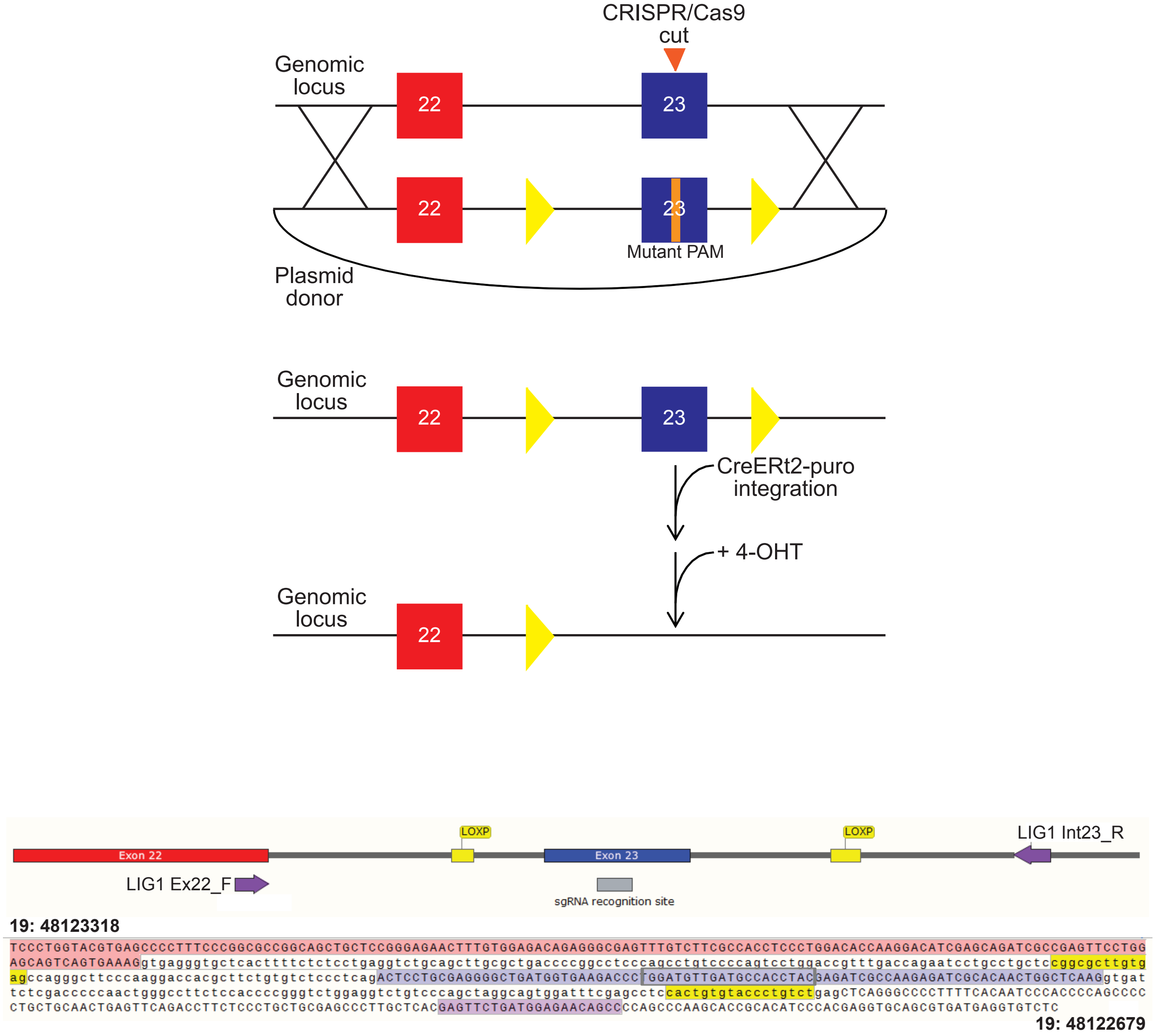

b

WT ACTCCTGCGAGGGGCTGATGGTGAAGACCCTGGATGTTGATGCCACCTACGAGATCGCCAAGAGATCGCACAACTGGCTCAAGgtgattctcgacccccaactgggccttctccaccccgggtctggaggtctgtcccagcta

Null ACTCCTGCGAGGGGCTGATGGTGAAG – -AAGg – – – - cccccaactgggccttctccaccccgggtctggaggtctgtcccagcta

Null ACTCCTGCGAGGGGCTGATGGTGAAGAAGgcccccaactgggcc

Supplementary Figure 4

c (i)

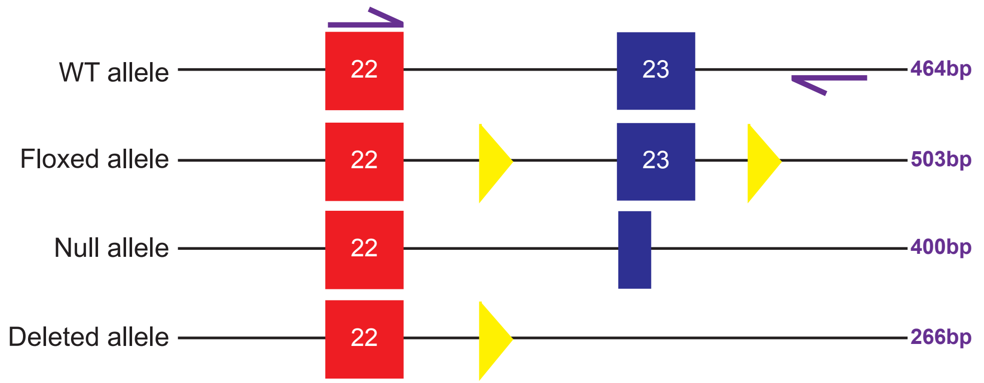

(ii)

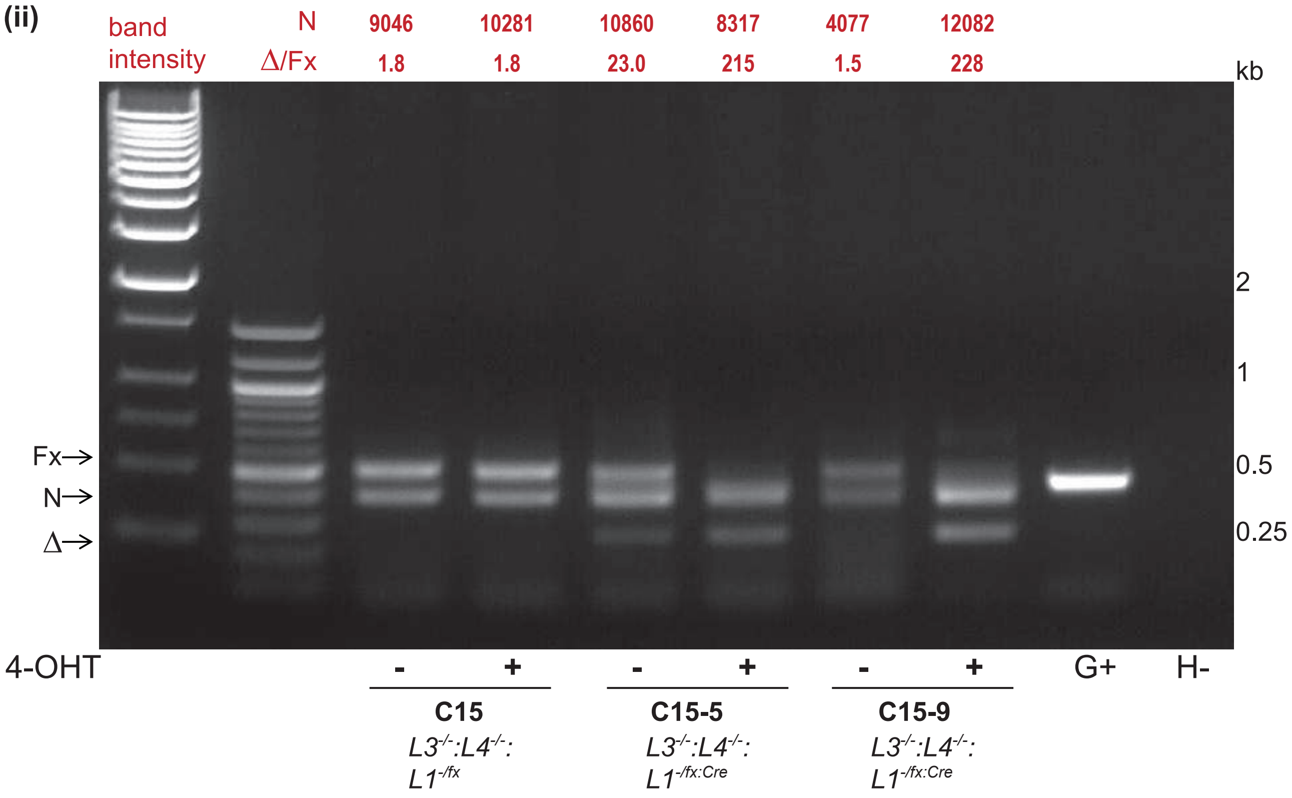

(iii)

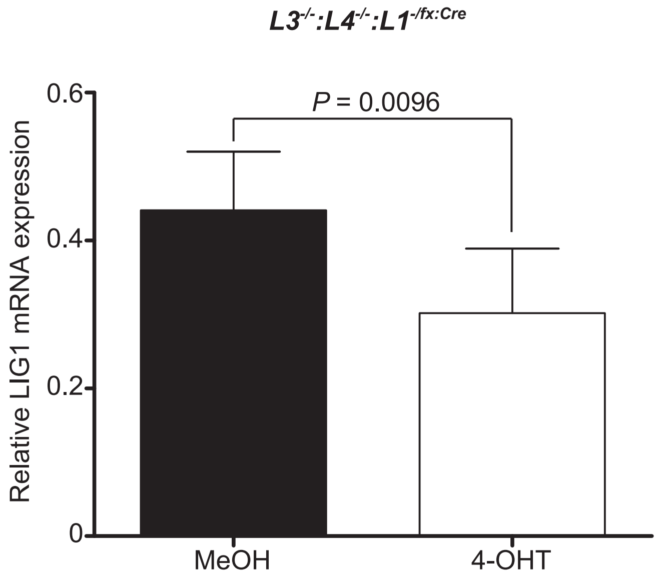

Supplementary Figure 4

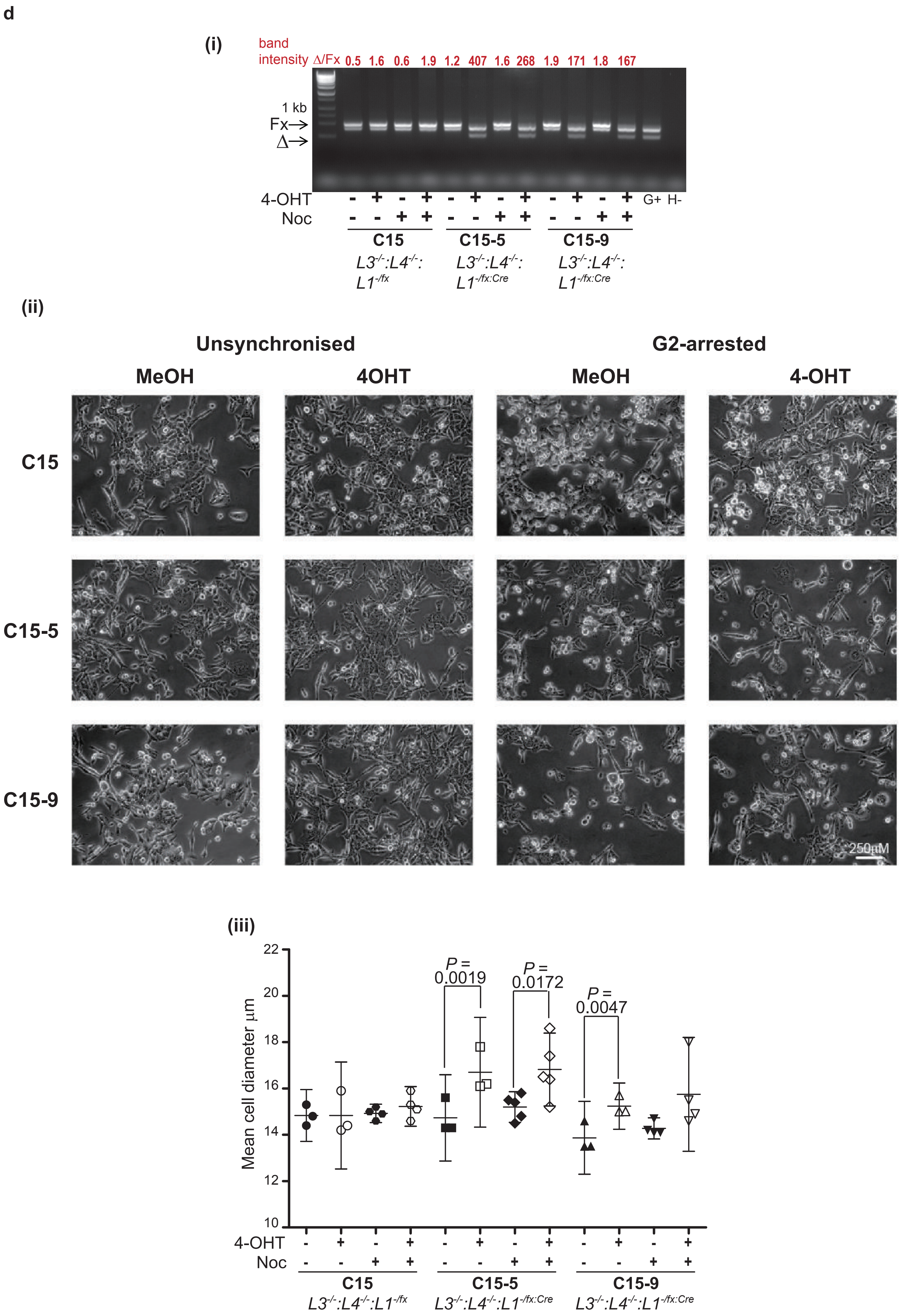

Supplementary Figure 5

(i) 21q-21q asymmetric fusion with microhomology

Δ35bp Δ2156bp

AGCCACGACAATGCCAGCAAGAGGGCCCGGCACTGTGCCCAGCTACCTCTCTCGACACCAAG

Δ82bp *microhomology* Δ2136bp

(ii) 21q-21q asymmetric inverted fusion with microhomology

Δ300bp Δ1866bp

GTACAAAGATGGAAGATAACTTCATTGAAAATATATACCAGGTGTGCTATCTTTTTGTGGTTTTCATTTCTCTA

Δ336bp *microhomology* Δ1823bp

(iii) 21q-21q inverted fragmented fusion with microhomology, insertions and duplications

Δ1844bp Δ1601bp Δ1105bp Δ300bp

GTAAAGAGCTTATTTTGTGGTT...TCTCTTCCA*CCATCATA*CCATACCAGG...AT*GATGATT*GGGGCATCTCAAGAGAAGTTC

Δ342bp Δ353bp *insertion* Δ605bp *microhomology* Δ796bp

(iv) 16p-Chr11 inverted fragmented fusion

Chr11:118920873-929 Chr11:118921181-504

GTCGATGGCACCTGACTGCA..GACATGTT//CTCCGCATG...CACCCACA//GAAACACA...GGGAAGT//CAGTGTCCCCGAG

Δ1781bp Chr11:118922527-479 Chr11:118922039-21955
